# Discovery of anti-SARS-CoV-2 secondary metabolites from the heartwood of *Pterocarpus santalinus* using multi-informative molecular networking

**DOI:** 10.3389/fmolb.2023.1202394

**Published:** 2023-06-06

**Authors:** Andreas Wasilewicz, Julia Zwirchmayr, Benjamin Kirchweger, Denisa Bojkova, Jindrich Cinatl, Holger F. Rabenau, Judith M. Rollinger, Mehdi A. Beniddir, Ulrike Grienke

**Affiliations:** ^1^ Division of Pharmacognosy, Department of Pharmaceutical Sciences, Faculty of Life Sciences, University of Vienna, Vienna, Austria; ^2^ Vienna Doctoral School of Pharmaceutical, Nutritional, Sport Sciences, University of Vienna, Vienna, Austria; ^3^ Institute of Medical Virology, University Hospital Frankfurt, Frankfurt am Main, Germany; ^4^ Équipe Chimie des Substances Naturelles, BioCIS, Centre National de la Recherche Scientifique (CNRS), Université Paris-Saclay, Orsay, France

**Keywords:** molecular networking, *Pterocarpus santalinus*, sandalwood, analysis, natural product, SARS-CoV-2, high-performance counter-current chromatography, isolation

## Abstract

A pigment-depleted extract from the heartwood of *Pterocarpus santalinus* L. f. (PS-DE) showed promising anti-SARS-CoV-2 activity with an IC_50_ of 29.9 μg/mL in Caco-2-F03 cells. To determine the potential active constituents within the extract prior to isolation, multi-informative molecular network (MN) was applied. Therefore, the extract was separated by high-performance counter-current chromatography (HPCCC) into 11 fractions which were subsequently tested for anti-SARS-CoV-2 activity and analysed by UPLC-tandem mass spectrometry (MS^2^). The resulting MN combines the bioactivity data of the fractions with the MS^2^ data. The MN analysis led to the targeted isolation of seven compounds including one pterocarpan **(7)** reported for the first time as constituent of *P. santalinus* and four so far undescribed natural products (NPs) that belong to the compound classes of arylpropanes **(9)**, isoflavanones **(10)** coumestans **(16)** and 3-arylcoumarins **(17)**, respectively. In total, 15 constituents from the heartwood of *P. santalinus* and one synthetic isoflavonoid that is structurally related to the natural metabolites were tested for anti-SARS-CoV-2 activity. Thereby, the two pterocarpans (−)-homopterocarpin **(5)** and (−)-medicarpin **(2)**, the stilbene *(E)*-pterostilbene **(1)** and the isoflavonoid 7-O-methylgenistein **(11)** showed a distinct antiviral activity with IC_50_ values of 17.2, 33.4, 34.7, and 37.9 µM, respectively, and no cytotoxic effects against Caco-2-F03 cells (CC_50_ > 100 µM). In addition, a structure-activity relationship (SAR) was proposed indicating structural requirements of pterocarpans for anti-SARS-CoV-2 activity. The herein presented results support the implementation of multi-informative molecular networks as powerful tool for dereplication and targeted isolation of bioactive NPs.

## 1 Introduction

*Pterocarpus santalinus* L. f. (Fabaceae), also known as red sandalwood, grows as small tree native to southern India. The red-coloured heartwood is used in Indian Ayurvedic medicine to treat various ailments such as headache, fever and chronic inflammatory disorders including chronic bronchitis (Dahat et al., 2021). In Europe, the heartwood is occasionally included in herbal detox tea preparations and as brightening agent (Wichtl, 2002).

In recent years, several *in vitro* studies have reported anti-inflammatory properties for extracts and constituents of *P. santalinus* heartwood using different cell model systems (Cho et al., 2001; Wu et al., 2011a; Wu et al., 2011b; Ham et al., 2019; Natalia et al., 2021; Zwirchmayr et al., 2023). Moreover, a heartwood extract also showed promising anti-influenza activity (IC_50_ of 12 μg/mL against influenza virus A in MDCK cells (CC_50_ of 54 μg/mL)) (Grienke et al., 2018). Two constituents from *P. santalinus* heartwood belonging to different compound classes were reported to have antiviral effects against coronaviruses (CoV). The lignan savinin exerted potent anti-SARS-CoV activity in Vero E6 cells with an EC_50_ in the low micromolar range (Wen et al., 2007). The activity of savinin was explained by the competitive inhibition of the SARS-CoV main protease (M^pro^, 3CL protease) which is essential for viral replication. Recently, the stilbene pterostilbene (**1**) has shown anti-SARS-CoV-2 activity in Vero E6 cells and in primary human bronchial epithelial cells under air-liquid interface cell culture conditions (ter Ellen et al., 2021). To date, no studies investigated the anti-SARS-CoV-activity of multi-component mixtures from *P. santalinus* heartwood.

Molecular networking, introduced by (Watrous et al., 2012), has rapidly emerged as an established and efficient dereplication tool to streamline the isolation of natural products (NPs) of interest from complex mixtures (Fox Ramos et al., 2019; Beniddir et al., 2021). The principle of molecular networking is based on the alignment and visualization of tandem mass spectrometry (MS^2^) data of biological samples such as extracts, fractions or pure compounds in a molecular network (MN) (Aron et al., 2020). Thereby, detected compounds are presented as “nodes.” The nodes of structurally related compounds with similar MS^2^ spectra are connected by “edges,” leading to the formation of molecular families. By comparison of the MS^2^ data from the MN with reference MS^2^ spectra data, nodes of known compounds can be annotated. These structural annotations can be used as starting point to annotate neighbour nodes via the calculation and interpretation of mass differences as well as literature. In recent years, the integration of bioactivity data into MNs has been successfully applied to identify bioactive NPs prior to their isolation (Olivon et al., 2017; Nothias et al., 2018). Thereby, the arrangement of structural and biological data in a MN gives certain advantages for the isolation of bioactives compared to classical bioassay-guided fractionation. While the targeted isolation of unknown bioactives can be prioritized, the re-isolation of NPs with already known bioactivity can be avoided.

Due to i) the traditional use against cold-related symptoms, ii) the described anti-inflammatory and anti-influenza properties, and iii) the reported anti-SARS-CoV activity of known constituents, the heartwood of *P. santalinus* was considered as promising natural source for the search of NPs with so far unknown anti-SARS-CoV-2 activity. In this study, a multi-informative MN was applied for the targeted isolation of NPs with anti-SARS-CoV-2 activity from the heartwood of *P. santalinus*.

## 2 Results and Discussion

### 2.1 Assessment of anti-SARS-CoV-2 activity of *Pterocarpus santalinus* heartwood

A crude extract (PS-E) and a pigment-depleted extract (PS-DE) were prepared from the *P. santalinus* heartwood. The anti-SARS-CoV-2 activity of PS-E and PS-DE was first evaluated with a pre-screening assay which is based on the measurement of caspase 3/7 activity of cells in response to SARS-CoV-2 infection (Bojkova et al., 2023). This assay represents an indirect readout method based on the measurement of the virus-induced caspase 3/7 activity of cells 48 h after SARS-CoV-2 infection. The results were then confirmed by means of a spike protein immunostaining assay in which the amount of spike protein was quantified 24 h after SARS-CoV-2 infection. For both assays, the SARS-CoV-2 strain G614 and Caco-2-F03 cells were used. The crude extract PS-E showed promising activity in the pre-screening assay but showed no activity in the spike immunostaining assay (IC_50_ > 100 μg/mL). The pigment-depleted extract PS-DE was determined as active in both assays showing an IC_50_ value of 29.9 μg/mL in the spike immunostaining assay. PS-DE was separated by high-performance counter-current chromatography (HPCCC+) and pooled into eleven fractions (F1—F11) according to TLC fingerprints (Supplementary Figure S1) (Zwirchmayr et al., 2023). All fractions were tested for anti-SARS-CoV-2 activity (Table 1). F2—F9 showed distinct activity in both assays (>60% of caspase 3/7 inhibition at 50 μg/mL and IC_50_ < 50 μg/mL in the spike immunostaining assay), F10 showed weak activity in the spike immunostaining assay. F1 and F11 were inactive in the pre-screening assay and were not further tested. Based on these findings, it was assumed that the *P. santalinus* heartwood extracts contain constituents with anti-SARS-CoV-2 activity but also metabolites that interfere with the used pre-screening assay. A false positive effect was confirmed for the crude extract PS-E. Other compounds have been already reported to give similar false positive effects in the used assay, i.e., the caspase inhibitor emricasan supressed SARS-CoV-2-induced caspase 3/7 activity but did not affect SARS-CoV-2 replication (Bojkova et al., 2023).

**TABLE 1:** Anti-SARS-CoV-2 activity and Caco-2-F03 cell cytotoxicity of *Pterocarpus santalinus* heartwood extracts and fractions.

	Anti-SARS-CoV-2 activity		Cytotoxicity	Selectivity index
	Caspase assay (at 50 μg/mL) [% inhibition]	Spike assay (IC_50_ ± SD) [µg/mL]	Caco-2-F03 (CC_50_ ± SD) [µg/mL]	CC_50_/IC_50_
PS-E	87.6	>100	>100	>1
PS-DE	121.4	29.9 ± 0.5	>100	>3.4
F1	33.3	n. t.	n. t.	-
F2	101.8	4.4 ± 0.6	>100	>22.6
F3	104.3	9.3 ± 2.6	>100	>10.7
F4	107.4	16.3 ± 1.0	46.2 ± 0.6	2.8
F5	115.4	10.4 ± 1.8	40.8 ± 1.4	3.9
F6	113.1	22.7 ± 0.9	>100	>4.4
F7	115.0	22.4 ± 4.7	66.0 ± 1.6	2.9
F8	60.2	35.2 ± 5.1	>100	>2.8
F9	99.3	12.6 ± 1.5	>100	>7.9
F10	101.1	55.4 ± 6.9	67.4 ± 16.1	1.2
F11	53.9	n. t.	n. t.	-
remdesivir (pos. control)	93.4 (at 10 µM)	0.17 ± 0.01 µM	>10 µM	>58.8

n. t. = not tested.

### 2.2 Molecular network generation and mapping

**FIGURE 1:**
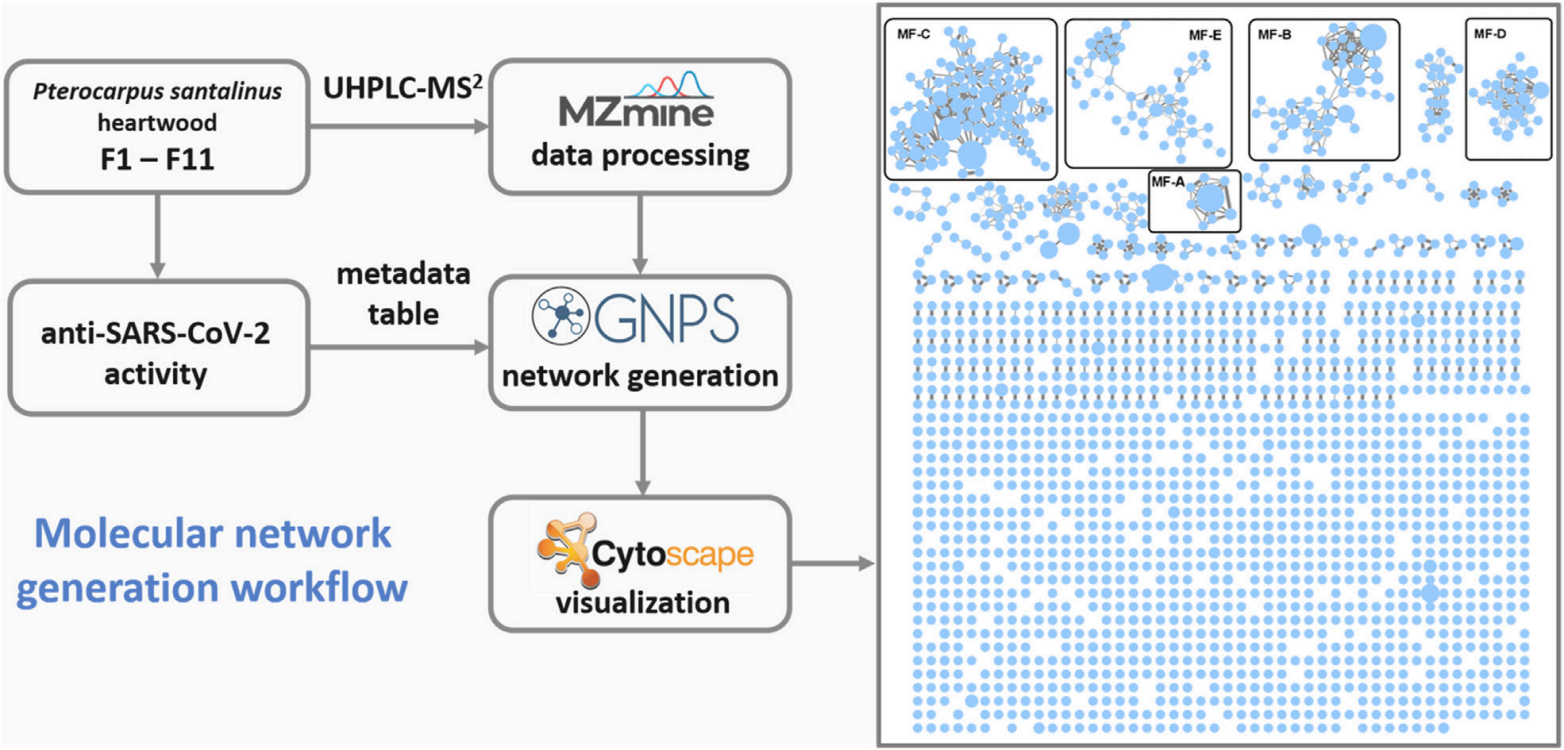
Workflow used for the generation of the global molecular network based on UHPLC-MS^2^ analyses of fractions F1-11. MF, molecular family.

**FIGURE 2:**
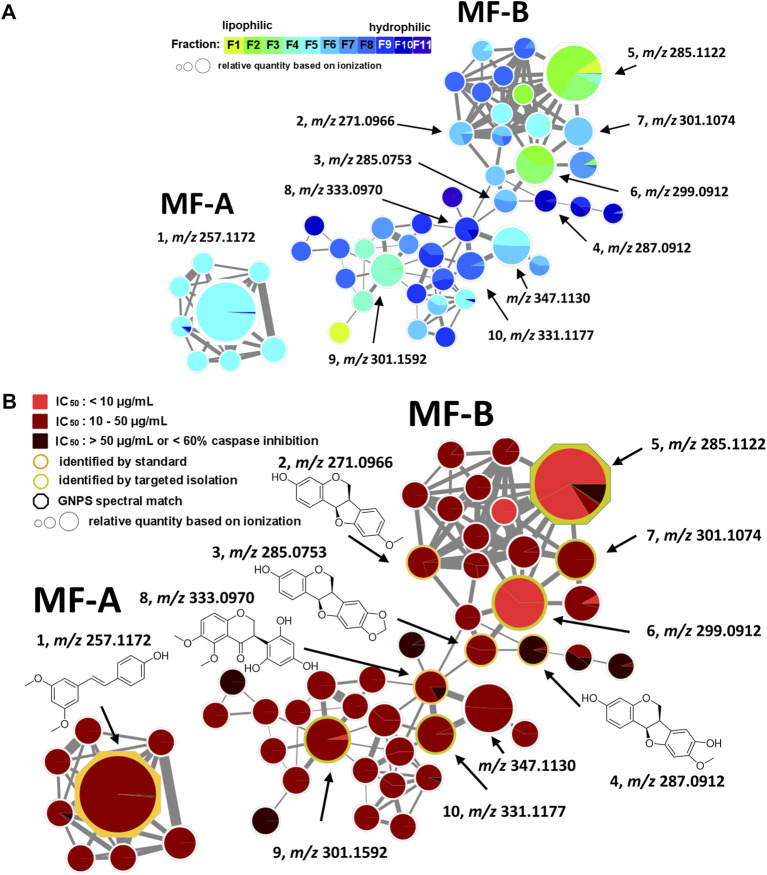
Molecular family A (MF-A) with compound **1** and molecular family B (MF-B) showing compounds **2**–**10**. **(A)** MF-A and MF-B with fraction-based mapping indicating the distribution of the compounds over F1—F11. **(B)** MF-A and MF-B with bioactivity-based mapping displaying the presence of the compounds in active, moderately active and inactive fractions. The chemical structures are shown for the identified compounds.

Each fraction (F1—F11) was subjected to UHPLC-MS^2^ (Supplementary Figures S2, S3). The generated MS^2^ data along with the bioactivity data (metadata table) were then uploaded to GNPS for MN generation (Aron et al., 2020). Figure 1 shows the entire MN. In order to visualize the distribution of the constituents over the fractions, a colour scale was used. Thereby, lipophilic fractions are coloured in yellow and green, while hydrophilic fractions are depicted in different shades of blue (Figure 2A). For the visualisation of bioactivity, the fractions were divided into three color-coded activity levels. Fractions with an IC_50_ < 10 μg/mL in the spike immunostaining assay were determined as active (coloured in fiery red) while fractions with an IC_50_ from 20 to 50 μg/mL in the spike immunostaining assay were rated as moderately active (coloured in ruby red). Fractions with IC_50_ > 50 μg/mL in the spike immunostaining assay or caspase 3/7 inhibition of <60% at 50 μg/mL were classified as inactive (coloured in black red). Hence, fractions F2 and F3 were categorized as active, F4—F9 were determined as moderately active and F1, F10 and F11 were classified as inactive. The size of the nodes reflects the relative quantity of a detected compound based on MS ionization (the higher the intensity, the bigger the node). The ionization rate of most of the constituents aligns well with the detected abundance in the UPLC-ELSD chromatograms (Supplementary Figures S4–S7). Structural network annotation was performed by comparison with i) previously isolated compounds from *P. santalinus* heartwood (orange node border) (Zwirchmayr et al., 2023), ii) GNPS spectra library (hexagon-shaped node) and iii) literature search (red node border).

### 2.3 Molecular network analysis and targeted isolation

The generated MN consists of 1,957 nodes including 23 molecular families (MF; >3 nodes). The nodes of five MFs (MF-A—MF-E) were explored in detail (Supplementary Table S1). Compound **1** [(*E*)-pterostilbene, *m/z* 257.1172 [M + H]^+^] with known anti-SARS-CoV-2 activity was found in MF-A. According to UPLC-ELSD, it is the major constituent of the moderately active fraction F5 (Figure 2). Notably, the lignan savinin, the second known constituent from *P. santalinus* heartwood with reported anti-SARS-CoV activity (Wen et al., 2007), could not be detected in any extract or fraction. Hence, it was quickly determined that the heartwood contained additional NPs with so far unknown anti-SARS-CoV-2 activity.

**FIGURE 3:**
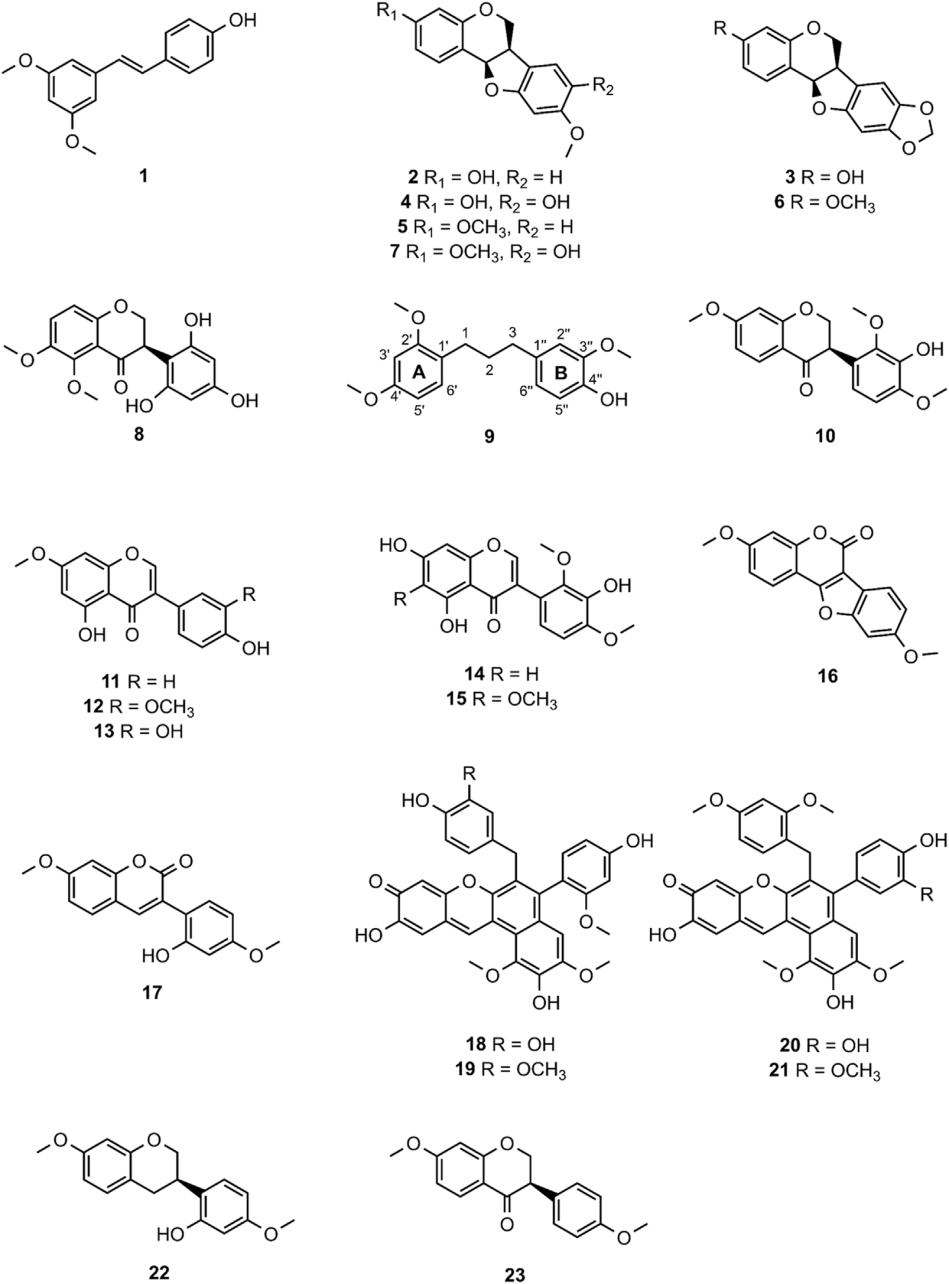
Chemical structures of compounds **1**—**23**.

MF-B shows a dumbbell-shaped structure. The top part of MF-B consists of compounds of the structure class of pterocarpans such as **2**–**4**. Compounds **2** [(−)-medicarpin, *m/z* 271.0966 [M + H]^+^], **3** [(−)-maackiain, *m/z* 285.0753 [M + H]^+^] and **4** [(−)-3,8-dihydroxy-9-methoxypterocarpan, *m/z* 287.0912 [M + H]^+^] were detected in F6, F7 and F10, respectively. Compounds **5**–**7**, which were all connected to each other and were the largest nodes in the top part of MF-B, remained unannotated after comparison with the previously isolated compounds. Compound **5** (*m/z* 285.1122 [M + H]^+^), annotated by the GNPS spectra library as homopterocarpin, and compound **6** (*m/z* 299.0912 [M + H]^+^) were of highest interest due to their presence in the active fractions F2 and F3 as major constituents determined by UPLC-ELSD. It should be noted that compound **5** was also detected in the inactive fraction F1 as indicated by the black red part of the chart. However, its relative abundance in F1 estimated by MS is overrated, since the major constituents of this fraction, presumably triterpenes, are difficult to ionize. Compound **7** (*m/z* 301.1074 [M + H]^+^) was found as major constituent in the moderately active fraction F6. Targeted isolation followed by NMR analysis and optical rotation experiments confirmed that (−)-homopterocarpin (**5**), (−)-pterocarpin (**6**) and (−)-8-hydroxyhomopterocarpan (**7**) belong to the structure class of pterocarpans (Figure 3). Compounds **5** and **6** are well-known constituents from *P. santalinus*, while compound **7** has been previously isolated from *Pterocarpus soyauxii* (Bezuidenhoudt et al., 1987). In the bottom part of MF-B, the isoflavanone **8** [(−)-pterosantalin C, *m/z* 333.0970 [M + H]^+^] was the only annotated compound. The three largest nodes in the bottom part of MF-B, the compounds **9** (*m/z* 303.1592 [M + H]^+^), **10** (*m/z* 331.1177 [M + H]^+^) and *m/z* 347.1130 [M + H]^+^, were investigated in detail. Compound **9** was the major component in F4 and was selected for targeted isolation. The ^1^H NMR spectrum of compound **9** showed six signals of aromatic protons [δ_H_ 6.41 (dd, *J* = 8.2, 2.4), δ_H_ 6.44 (d, *J* = 2.3), δ_H_ 6.69 (d, *J* = 1.7), δ_H_ 6.69 (dd, *J* = 8.4, 1.9) δ_H_ 6.82 (d, *J* = 8.5) δ_H_ 7.02 (d, *J* = 8.2)], three signals assignable to methoxy groups [δ_H_ 3.79 (6H, s), δ_H_ 3.87 (3H,s)] and three signals of methylene groups [δ_H_ 1.85 (2H, m), δ_H_ 2.58 (4H, t, *J* = 7.7)]. These chemical shifts corresponded well with signals determined for NPs with a diphenylpropane scaffold (Pan et al., 2010). The substitution pattern of ring A was assigned by the HMBC correlation from H-6′ (δ_H_ 7.02) to C-1 (δ_C_ 29.40), C-2′ (158.46) and C-4′ (δ_C_ 159.17), while the substitution pattern of ring B was determined by the HMBC correlation from H-5′′ (δ_H_ 6.82) to C-1′′ (δ_C_ 134.83), C-3′′ (δ_C_ 146.38), C-4′′ (δ_C_ 143.59) and C-6′′ (δ_C_ 121.09). Eventually, compound **9** was identified as new NP belonging to the class of arylpropanes (i.e. 1,3-diphenylpropanes) and was named pterosantalin D (Table 2). Compound **10** in F8 and *m/z* 347.1130 in F5 were only detected in traces by UPLC-ELSD and were not further considered for isolation. However, compound **10** was co-isolated during targeted isolation of compound **16**. It was identified as an isoflavanone which was previously known as synthetic compound and was named (−)-pterosantalin E (**10**) (Maranduba et al., 1979). Due to direct connections to compounds **8** and **10,** *m/z* 347.1130 was supposed to be an isoflavonone substituted by three methoxy and two hydroxy groups with the molecular formula of C_18_H_18_O_7_. The relatively large node size of *m/z* 347.1130 can be explained by its higher ionization rate in comparison to the major constituent, compound **1**, in F5.

**TABLE 2:** ^1^H (500 MHz) and ^13^C (125 MHz) NMR Data of Pterosantalin D (9) (CDCl_3_, δ in ppm).

Position	δ_H_, mult (*J* in Hz)	δ_C_	HMBC correlations
1	2.58, t (7.7)	29.40	C-2, C-3, C-1′, C-2′, C-6′
2	1.85, m	31.98	C-1, C-3, C-1′, C-1″
3	2.58, t (7.7)	33.51	C-1, C-2, C-1″, C-2″, C-6″
1′	—	123.28	—
2′	—	158.46	—
3′	6.44, d (2.3)	98.59	C-1′, C-2′, C-4′, C-5′
4′	—	159.17	—
5′	6.41, dd (8.2, 2.4)	103.79	C-1′, C-2′, C-3′, C-4′
6′	7.02, d (8.2)	130.06	C-1, C-2′, C-4′
1″	—	134.83	—
2″	6.69, d (1.7)	111.09	C-3, C-1″, C-3″, C-6″
3″	—	146.38	—
4″	—	143.59	—
5″	6.82, d (8.5)	114.17	C-6″
6″	6.69, dd (8.4, 1.9)	121.09	C-2″, C-4″, C-6″
2′-OMe	3.79, s	55.40	C-2′
4′-OMe	3.79, s	55.49	C-4′
3″-OMe	3.87, s	55.99	C-3″

**FIGURE 4:**
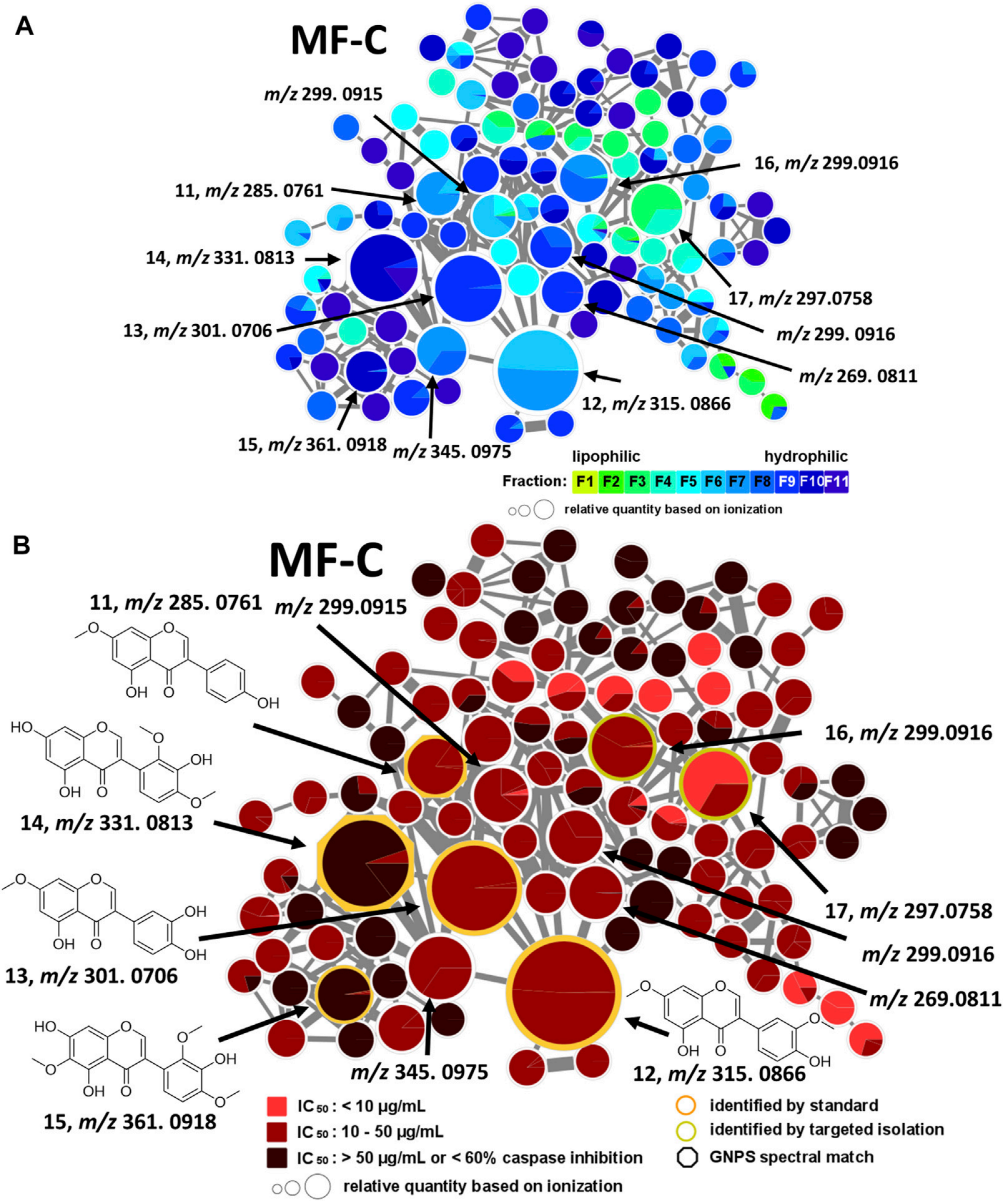
Molecular family C (MF-C) including compounds **11**—**17**. **(A)** MF-C with fraction-based mapping indicating the distribution of the compounds over F1—F11. **(B)** MF-C with bioactivity-based mapping displaying the presence of the compounds in active, moderately active and inactive fractions. The chemical structures are shown for the identified compounds.

MF-C mostly contains isoflavones such as compounds **11**—**15** (Figure 4) which have been previously isolated from *P. santalinus* heartwood (Zwirchmayr et al., 2023). Compounds **11** [7-*O*-methylgenistein, *m/z* 285.0761 [M + H]^+^] and **12** [7,3′-di-*O*-methylorobol, *m/z* 315.0666 [M + H]^+^] were found in F7 and were the major constituents of this fraction according to UPLC-ELSD analysis. Compound **13** [santal, *m/z* 301.0706 [M + H]^+^] was detected as the most abundant compound in fraction F9. Compounds **14** [khrinone C, *m/z* 331.0813 [M + H]^+^] and **15** [pterosantalin B, *m/z* 361.0918 [M + H]^+^] were mostly found in F10. Four additional nodes of minor compounds (m/z 269.0811 [M + H]^+^, 299.0915 [M + H]^+^ (RT: 6.30 min), 299.0916 [M + H]^+^ (RT: 5.82 min), 345.0975 [M + H]^+^ were putatively annotated as isoflavones which differ in the number and position of hydroxy and methoxy groups. Compounds **16** (*m/z* 297.0758 [M + H]^+^) and **17** (*m/z* 299.0916 [M + H]^+^) were not dereplicated prior to isolation. Due to their occurrence in active and moderately active fractions, compounds **16** (in F3 and F4) and **17** (in F8) were both selected for targeted isolation. Since neither compound was directly connected to the annotated isoflavonoids, it was expected that compound **16** and **17** belong to structurally related compound classes. Eventually, isolation and subsequent NMR analysis identified compound **16** as a coumestan named 3,9-*O*-dimethylcoumestrol and **17** as a 3-arylcoumarin named pterosantalin F. This is the first report of those previously synthesized compounds as genuine compounds (Harper et al., 1969; Naik et al., 2017).

**FIGURE 5:**
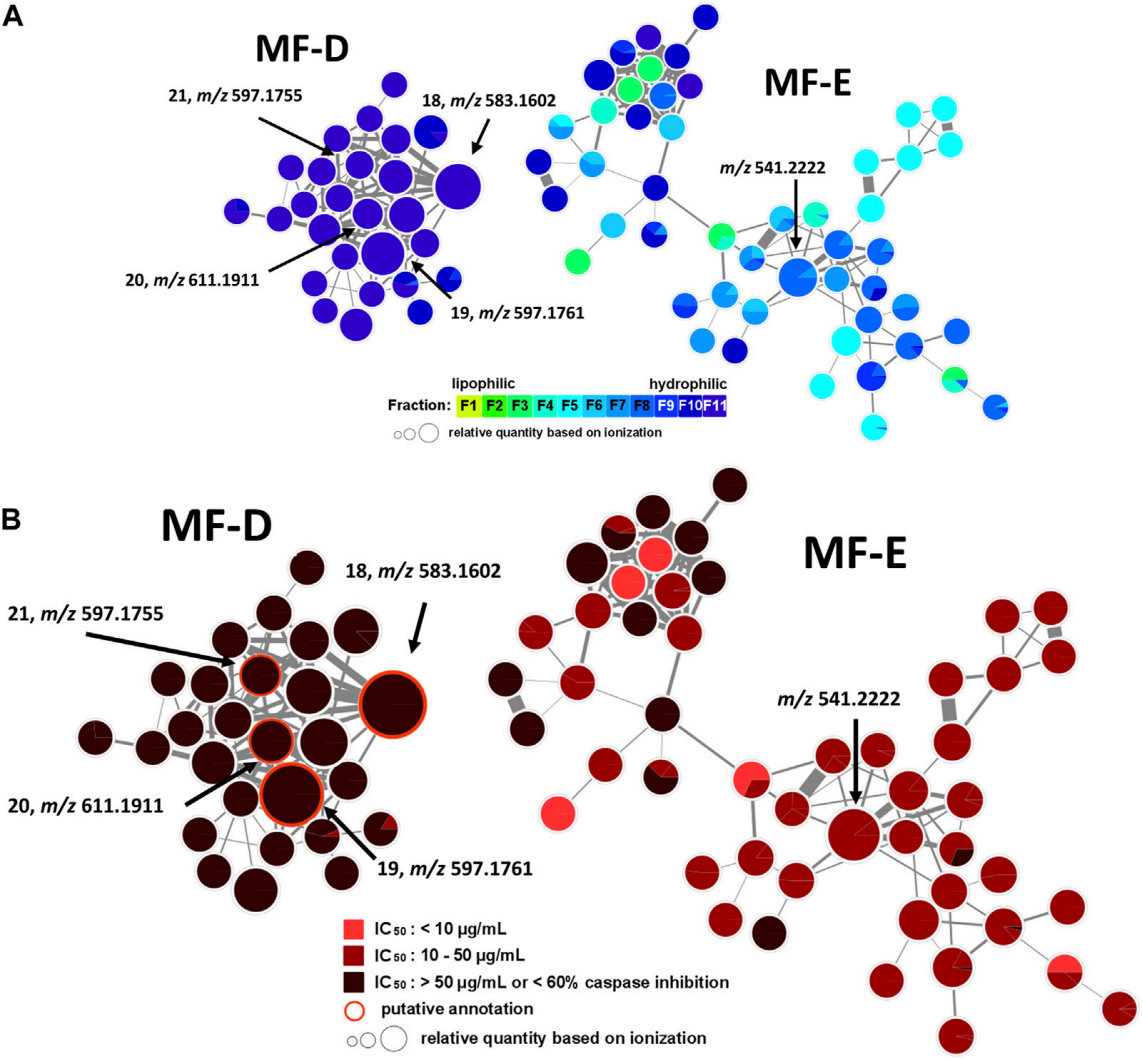
Molecular family D (MF-D) with compounds **18**—**21** and molecular family E (MF-E). **(A)** MF-D and MF-E with fraction-based mapping indicating the distribution of the compounds over F1—F11. **(B)** MF-D and MF-E with bioactivity-based mapping displaying the presence of the compounds in active, moderately active and inactive fractions. The chemical structures of identified compounds are shown.

In addition, MF-D that only consists of metabolites from the inactive F11 was roughly characterised (Figure 5). Thereby, four major pigments reported for *P. santalinus* heartwood, compound **18** (santalin A, *m/z* 583.1602 [M + H]^+^), **19** (santalin B, *m/z* 597.1761 [M + H]^+^), **20** (santarubin A, *m/z* 611.1911 [M + H]^+^), and **21** (santarubin B, *m/z* 597.1755 [M + H]^+^) were tentatively annotated. F11 was the fraction with by far the highest determined yield (Supplementary Table S2). Hence, the lack of bioactivity of the crude extract PS-E in the spike assay could likely be due to the high content of non-active pigments occurring as bulk constituents.

The major constituent of F8 according to UPLC-ELSD was found in MF-E. The relatively small node size with *m/z* 541.2222 can be explained by the poor ionization rate. Since all nodes from MF-E remained unannotated, only vague assumptions concerning the potential compound class were made. If *m/z* 541.2222 is assumed to be an [M + H]^+^ ion, and considering the high abundance of isoflavonoids and related compound classes in the heartwood, this compound could possibly be a bi-isoflavonoid with the molecular formula C_30_H_20_O_10_. Bi-isoflavonoids have been previously found in other plants belonging to the Fabaceae family such as in *Podocarpium* and *Dalbergia* species (Yahara et al., 1985; Ma et al., 2013). Targeted isolation of m/z 541.2222 was conducted but could not be achieved due to the presence of a potential isomer. After unsuccessful separation attempts using orthogonal techniques such as HPCCC+ and size-exclusion chromatography (SEC), the isolation was not further pursued.

### 2.4 Anti-SARS-CoV-2 activity of pure compounds

In total, 15 compounds were subjected to the anti-SARS-CoV-2 spike immunostaining assay (Table 3) (**1**–**7**, **9**, **11**–**14**, **16, 22**, **23**). The minor compounds **8** and **17** as well as compound **10** were not tested since only low yields were isolated which were insufficient for testing. Based on the network analysis, compounds **5** and **6** were expected to show distinct bioactivity, compounds **1**, **2**, **3**, **7**, **9**, **11**, **12**, **13** and **16** were predicted to be moderately active, and compounds **4** and **14** were supposed to be inactive. The activity of compound **16** could not be assessed since it was only a minor compound in F8. The isoflavane **22** ((−)-isosativan) was not present in the MN but was found in F4 and was selected for testing due to its structural resemblance to compound **5**. In addition, the synthetic compound **23** ((+)-7,4′-di-*O*-methyldihydrodaidzein) which is the isoflavanone structural analogue of compound **5** was included.

**TABLE 3:** Anti-SARS-CoV-2 activity and Caco-2-F03 cell cytotoxicity of pure compounds.

	MN Analysis		*In vitro* testing		
Compound	found in	Activity classification	Anti-SARS-CoV-2 activity (IC_50_ ± SD) [µM]	Cytotoxicity (CC_50_ ± SD) [µM]	Selectivity index (CC_50_/IC_50_)
**1**, (*E*)-pterostilbene	F5	moderate	34.7 ± 1.6	>100	>2.9
**2**, (−)-medicarpin	F6	moderate	33.4 ± 9.7	>100	>3.0
**3**, (−)-maackiain	F7	moderate	>100	>100	1
**4**, (−)-3,8-dihydroxy-9-methoxypterocarpan	F10	inactive	>100	>100	1
**5**, (−)-homopterocarpin	(F1), F2, F3	active	17.2 ± 1.9	>100	>5.8
**6**, (−)-pterocarpin	F2, F3	active	>100	>100	1
**7**, (−)-8-hydroxyhomo-pterocarpan	F6	moderate	>100	>100	1
**9**, pterosantalin D	F4	moderate	>100	>100	1
**11**, 7-*O*-methylgenistein	F7	moderate	37.9 ± 5.4	>100	>2.6
**12**, 7,3′-di-*O*-methylorobol	F7	moderate	>100	>100	1
**13**, santal	F9	moderate	74.2 ± 15.4	>100	>1.4
**14**, khrinone C	F10	inactive	>100	>100	1
**16**, 3,9-di-*O*-methylcoumestrol	F3, F4	n. d.	>100	>100	1
**22**, (−)-isosativan	F4*	—	40.8 ± 1.8	>100	>2.5
**23**, (+)-7,4′-di-*O*-methyldihydrodaidzein	n. f.	—	53.3 ± 6.2	>100	>1.9
remdesivir (pos. control)	—	—	0.17 ± 0.01	>10	>58.8

n. f. not found, n. d. not determined, *not found in MN.

Eventually, compound **5** was determined as the most active constituent with an IC_50_ of 17.2 µM. Moreover, compounds **1** (IC_50_ of 34.7 µM), **2** (IC_50_ of 33.4 µM), **11** (IC_50_ of 37.9 µM), **22** (IC_50_ of 40.8 µM) and **23** (IC_50_ of 53.3 µM) showed moderate anti-SARS-CoV-2 activity. The determined activity of compound **1** in this study is in line with the previously reported activity (ter Ellen et al., 2021). Compound **13** (IC_50_ of 74.2 µM) showed weak anti-SARS-CoV-2 activity. As expected, compounds **4** and **14** were determined as inactive as well as compounds **3**, **6**, **7**, **9**, **12** and **16** (IC_50_ > 100 µM). In summary, the bioactivities of F2 and F3 (containing compound **5**), F4 (containing compound **22**), F5 (containing compound **1**), F6 (containing compound **2**) and F7 (containing compound **11**) were pinpointed to specific constituents, while the activity of F8 remained elusive. The activity of the mother extract PS-DE was mainly attributed to compounds **1** and **5**, as they were determined to be the major constituents in the extract along with the inactive compounds **6**, **13** and the red-coloured pigments.

To evaluate the potential molecular target involved in the phenotypic activity, compound **5** with the strongest anti-SARS-CoV-2 activity was tested for SARS-CoV-2 main protease (M^pro^) inhibition using an enzyme-based FRET assay (Wasilewicz et al., 2023). However, compound **5** did not show any inhibitory activity against M^pro^ at 50 µM when compared to the DMSO control (data not shown, method in Supplementary Material). Hence, M^pro^ can be excluded as the molecular target for compound **5**.

### 2.5 Structure-activity relationship of pterocarpanoids and anti-SARS-CoV-2 activity

**FIGURE 6:**
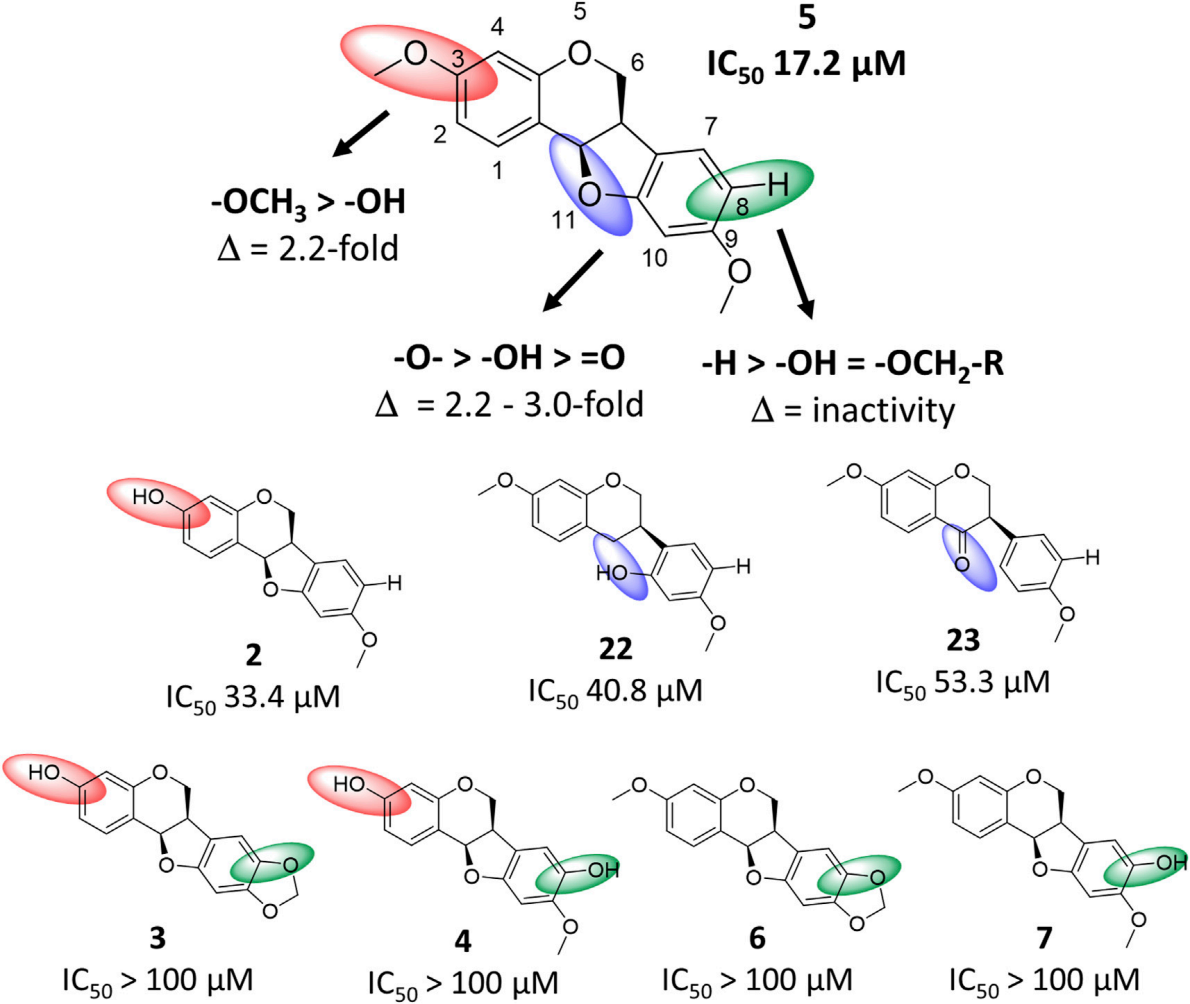
Structure-activity relationship chart of compound **5** and related compounds **2**–**4**, **6**, **7**, **22** and **23** for anti-SARS-CoV-2 activity. Structural changes compared to compound **5** at position C-3, C-8 and C-11 are depicted by red, green and blue spheres, respectively.

By comparing the bioactivity from the spike assay of the most active compound **5** with compounds **2**–**4**, **6**, **7**, **22** and **23**, a structure-activity relationship (SAR) for pterocarpan compounds was proposed (Figure 6). Thereby, three structural requirements were determined. The substitution of position C-3 by a methoxy group resulted in a 2.2-fold increase of bioactivity compared to the substitution with a hydroxyl moiety as seen in compound **2**. Furthermore, any type of position C-8 substitution led to a complete abolishment of bioactivity as it was the case for compounds **3**, **4**, **6** and **7**. Finally, the ether group in position C-11 plays an essential role for bioactivity. Compound **11** possesses a 3.0-fold higher activity than its corresponding isoflavone **23** with a keto group and is 2.2-fold more active than the isoflavane **22** whose hydroxy group aligns well with the ether group according to 3D structural alignment (Supplementary Figure S8).

In the present study, bioactivity-informed molecular networking was successfully applied to identify NPs with anti-SARS-CoV-2 activity from *P. santalinus* heartwood prior to isolation. Instead of laborious iterative fractionation and testing cycles needed for bioassay-guided isolation approaches, seven compounds were isolated in a target-oriented manner. Molecular networking not only revealed compounds with potential bioactivity but also facilitated the dereplication of known major constituents from the heartwood. Based on the structural annotations, MF-B and MF-C were well characterised revealing the heartwood as a rich source of isoflavonoids and pterocarpan compounds. However, MFs without structurally annotated compounds such as MF-E are difficult to explore. Since there are several MFs without any structural annotations, the heartwood of *P. santalinus* remains a promising source to find so far undescribed NPs.

The findings support the ethnopharmacological use of *P. santalinus* heartwood in Ayurvedic medicine against cold-related symptoms. Compound **5** (IC_50_ of 17.2 µM) was determined as the most active constituent and is assumed to be mainly responsible for the bioactivity shown by the investigated pigment-depleted extract PS-DE (IC_50_ of 29.9 μg/mL) alongside compound **1** (IC_50_ of 34.7 µM). In addition, compounds **2**, **11**, **22** and **23** have shown moderate activity ranging from 33.4 to 53.3 µM. All bioactive NPs were well tolerated by Caco-2-F03 cells (CC_50_ > 100 µM). For the evaluation of the anti-SARS-CoV-2 activity, an *in vitro* phenotypic assay was performed. Compound **5** had no inhibitory activity against the commonly tested SARS-CoV-2 target M^pro^. Recent results from time-of-addition assays with compound **1** suggested that the anti-SARS-CoV-2 activity occurs in post-infection conditions within the cells (ter Ellen et al., 2021). The exact mechanism of action and potential molecular targets of compounds discovered in this study are still unknown and require further investigations. Nevertheless, the chemical scaffold of pterocarpan compounds presents a promising starting point for the development of more potent compounds with anti-SARS-CoV-2 activity.

## 3 Material and methods

### 3.1 General experimental procedures

The UPLC-ELSD analysis was performed on a Waters Acquity UPLC H-class system comprising a quaternary solvent manager, sample manager, column manager, PDA detector and ELSD. The instrument was controlled by the software Empower 3. An HSS T3 column (1.7 µm, 2.1 × 100 mm, Waters) was used at 40°C as stationary phase, while the mobile phase consisted of water (A) and acetonitrile (B). The flow rate was set to 0.25 mL/min. The following gradient was used: 30% B isocratic for 0.5 min, 30%–45% B in 1.5 min, 45%–50% B in 1.5 min, 50%–55% B in 8.5 min, 55%–98% B in 0.1 min, 98% B for 5.9 min.

Flash chromatography (FC) was performed on PuriFlash 4250 from Interchim equipped with a PDA detector and ELSD and was controlled by the Interchim software. High-performance counter-current chromatography (HPCCC) was performed using a HPCCC+ device (Zwirchmayr et al., 2023). Therefore, the PuriFlash instrument was hyphenated to a Spectrum HPCCC instrument from Dynamic Extraction Ltd., whereby the HPCCC device replaces a solid stationary column. An Accel 500 LC chiller from Thermo Scientific was used for cooling.

TLC was carried out using Merck silica gel 60 PF254 plates as stationary phase. The mobile phase consisted of chloroform:ethyl acetate:formic acid (8:1:1). Detection was conducted under visible light, UV_254_ and UV_366_ before and after derivatisation with vanillin/sulfuric acid (5% in methanol).

SEC was performed with Sephadex LH-20 as stationary phase and methanol as mobile phase (column size: 100 cm × 2 cm).

NMR experiments were conducted on a Bruker Advance 500 NMR spectrometer (UltraShield) equipped with a TCI Prodigy CryoProbe (5 mm, triple resonance inverse detection probe head). NMR spectra were analysed by using TopSpin 4.1.4.

The optical rotation was measured at 25°C in methanol or chloroform using an MCP 100 polarimeter.

### 3.2 Plant material

The *P. santalinus* heartwood was purchased from Kottas Pharma GmbH, Vienna, Austria (batch no. P16301836). A voucher specimen (JR-20190315-A1) is deposited at the Division of Pharmacognosy, Department of Pharmaceutical Sciences, University of Vienna, Austria.

### 3.3 Generation of PS-E and PS-DE

First, a heartwood crude extract (PS-E) was generated as previously described (Zwirchmayr et al., 2023). 1 kg of the dried, pulverized heartwood was defatted with 2 L of n-hexane for 3 days. Then, the remaining material was extracted three times with 4 L of dichloromethane for 3 days. The extraction procedure was repeated with methanol. The dichloromethane and methanol extracts were combined and dried under vacuum to obtain the extract PS-E. For the pigment depletion, liquid-liquid extraction (LLE) was performed using dichloromethane and 50% aqueous methanol (1:1, v/v) as biphasic solvent system. The LLE process of pigment depletion was chosen because it is considered as the most efficient method for the removal of bulk constituents (Picker et al., 2014). The solvent system was selected as the most suitable of six systems tested in preliminary trials monitored by TLC. Approximately 85 g of PS-E were separated by 16 cycles of LLE (500 mL of each phase). The combined dichloromethane phases were evaporated to dryness yielding 67.3 g of the pigment-depleted extract (PS-DE).

### 3.4 Generation of F1—F11

PS-DE fractionation was performed using two semi-preparative runs of HPCCC+ in normal phase mode at a flow rate of 6 mL/min and rotation speed of 1,600 rpm. About 800 mg of PS-DE was fractionated using the HEMWat biphasic solvent system, which consists of several mixtures with different proportions of hexane, ethyl acetate, methanol and water (Supplementary Table S3). The lower layer (LL) of HEMWat system 19 (300 mL) was used as the stationary phase, while the upper layers (UL) of HEMWat systems 19, 18, 17, 16 and 14 (250 mL of each layer) were successively used as mobile phase. For a stepwise gradient elution, the following procedure was used. When 200 mL of a mobile phase was consumed, the residual 50 mL were blended with 50 mL of the next mobile phase. After the consumption of 50 mL of this mixture, the remaining volume of the next mobile phase was added. 6 mL per fraction were collected. After the appearance of the first red coloured fractions, elution extrusion was performed using 50% aqueous methanol at a flow rate of 10 mL/min and a rotation speed of 200 rpm. The elution extrusion was collected as a whole. In total, 392 fractions were collected and analysed by TLC resulting in 11 pooled fractions (F1—F11), with the final fraction being the elution extrusion.

### 3.5 Targeted isolation of *Pterocarpus santalinus* constituents and pure compounds

A fractionation tree is provided in Supplementary Figure S9. Approximately 5 mg of F6 were subjected to SEC to yield three fractions (PS-DE_F6a-c). PS-DE_F6a was identified as compound **7** (0.73 mg; (−)-8-hydroxyhomopterocarpan) (Bezuidenhoudt et al., 1987).

PS-DE was fractionated using FC as described previousely (Zwirchmayr et al., 2023). Briefly, about 10 g of PS-DE were applied via dry load on a Silica HC 200 G column (stationary phase). Petroleum ether (A), dichloromethane (B) and methanol (C) were selected as mobile phase using the following gradient: 80% A/20% B isocratic for 13 min, 80% A/20% B—100% B in 3 min, 100% B isocratic for 16 min, 100% B—50% B/50% C in 45 min, 50% B/50% C—30% B/70% C in 1 min, 30% B/70% C isocratic for 15 min. The flow rate was set to 100 mL/min. The collected fractions were analysed by TLC resulting in four combined fractions PS-DE_A-D.

About 350 mg of PS-DE_A were fractionated by HPCCC+ in normal phase mode (flow rate of 6 mL/min, 1,600 rpm) using HEMWat system 23 (300 mL of LL, 1.8 L of UL) to yield three final fractions (PS-DE_A1-3). PS-DE_A2 was identified as compound **5** (235.89 mg; (−)-homopterocarpin (Bezuidenhoudt et al., 1987).

Approximately 250 mg of PS-DE_B were separated by HPCCC+ in normal phase mode (flow rate of 6 mL/min, 1,600 rpm) using HEMWat systems 23 (300 mL of LL, 400 mL of UL) and 19 (700 mL of UL) to yield eight fractions (PS-DE_B1-8). PS-DE_B3 and PS-DE_B5 were identified as compound **6** (0.8 mg (−)-pterocarpin) and the new compound **9** (2.07 mg; pterosantalin D (B-son Bredenberg and Shoolery, 1961). PS-DE_B4 was further subjected to SEC to yield two final fractions (PS-DE_B4a-b). PS-DE_B4b was identified as compound **16** (3.87 mg, 3,9-di-O-methylcoumestrol) (Harper et al., 1969).

About 1 g of PS-DE_C was fractionated by four runs of FC in reversed phase mode using a 15 µm C_18_ HQ 35 G column as stationary phase and water (A) and acetonitrile (B) as mobile phase. The following gradient was applied: 5% B isocratic for 10 min, 5%–30% B in 10 min, 30% B isocratic for 30 min, 30%–50% B in 30 min, 50% B isocratic for 15 min, 50%–98% B in 10 min, 98% B isocratic for 30 min. The flow rate was set to 15 mL/min. The collected fractions were analysed by TLC yielding in 10 fractions (PS-DE_C1-10). Approximately 250 mg of PS-DE_C5 were further separated by HPCCC+ in normal phase mode (flow rate of 6 mL/min, 1,600 rpm) using HEMWat systems 19 (300 mL of LL, 300 of UL), 18 (600 mL of UL), 17 (600 mL of UL) and 13 (120 mL of UL) resulting in six fractions (PS-DE_C5a-f). PS-DE_C5b was subjected to SEC and led to the isolation of compounds **10** (0.71 mg (−)-pterosantalin E) and **17** (0.52 mg, pterosantalin F) (Maranduba et al., 1979; Naik et al., 2017). Identity of all isolated compounds was assessed by 1D and 2D NMR, HR-MS and optical rotation.

Compounds **1**–**4**, **11**–**15** and **22** have been previously isolated from the heartwood of *P. santalinus* (Zwirchmayr et al., 2023). Compound **23** was obtained from Specs.

***Pterosantalin D* (9):** brownish, amorphous powder; ^1^H (CDCl_3_, 500 MHz) and ^13^C NMR (CDCl_3_, 125 MHz), see Table 2 and Supplementary Figures S10–S13; HRESIMS *m/z* 303.1592 [M + H]^+^ (calcd for C_18_H_23_O_4_^+^, 303.1518).

### 3.6 UHPLC-HRMS^2^ data generation

LC-ESI-HRMS^2^ analyses were performed on an Agilent 1290 Infinity II UHPLC coupled to a hybrid quadrupole time of flight (QTOF) mass spectrometer Agilent 6546 (Agilent Technologies) equipped with an ESI source, operating in positive ion mode. A BEH Waters Acquity C_18_ UPLC column (2.1 × 150 mm; 1.7 µm) was used, while water + 0.1% formic acid (A) and acetonitrile + 0.1% (B) were used as mobile phase. The flow rate was set to 0.5 mL/min. A linear gradient from 5% to 100% B in 12 min followed by 100% B for 4 min was used. Source parameters were set as follows: capillary temperature at 320°C, source voltage at 3,500 V, sheath gas flow rate at 11 L/min. MS scans were operated in full-scan mode from *m/z* 100 to 1,200 (0.1 s scan time) with a mass resolution of 67.000 at *m/z* = 922. A MS1 scan was followed by MS2 scans of the four most intense ions above an absolute threshold of 3,000 counts. Selected parent ions were fragmented at a collision energy fixed at 45 eV and an isolation window of 1.3 amu. The purine [M + H]^+^ ion (C_5_H_5_N_4_^+^, *m/z* = 121.0509) and the hexakis (1H,1H, 3H-tetrafluoropropoxy)phosphazene [M + H]^+^ ion (C_18_H_19_F_24_N_3_O_6_P_3_^+^, *m/z* = 922.0098) were used as internal lock masses. A permanent MS/MS exclusion list criterion was set to prevent oversampling of the internal calibrant. LC-UV and MS data acquisition and processing were performed using MassHunter Workstation software (Agilent Technologies).

### 3.7 MZmine 3 processing for feature-based molecular networking

The MS^2^ data files were converted from the .d format to the .mzZML format using MSConvert from ProteoWizard (Chambers et al., 2012). The .mzXML files were then processed using MZmine 3 (version 3.1.0-beta) (Schmid et al., 2023). For mass detection, a noise level of 1 × 10^4^ and 1 × 10^1^ was specified for MS^1^ and MS^2^, respectively. The ADAP chromatogram builder was used with the following settings: minimum group size of scans = 4, group intensity threshold = 1 × 10^4^, minimum highest intensity = 1 × 10^4^, *m/z* tolerance = 10 ppm (Schmid et al., 2023). Next, the ADAP chromatogram deconvolution module was employed with the following settings: S/N threshold = 23, minimum feature height = 1 × 10^4^, coefficient/area threshold = 10, peak duration length = 0.8 min, RT wavelet range = 0.0—0.08 (Myers et al., 2017). Isotopes were grouped by the ^13^C isotope filter, setting an *m/z* tolerance of 10 ppm, maximum charge of 1 and RT tolerance of 0.3 min. Peak alignment was performed by the join aligner module, selecting a *m/z* tolerance of 10 ppm, weight for *m/z* and RT of 50 and RT tolerance of 0.12 min. The peak list was gap-filled using the same RT and *m/z* range gap filler with a *m/z* tolerance of 10 ppm. The feature list row filter was applied which only kept peaks with *m/z* from 100.00 to 1,200.00 and RT from 0.04 to 16.00 min. The data from the resulting peak list was exported in .mgf and .csv format.

### 3.8 Generation of the metadata table

A metadata table was created using Notepad++ to incorporate the bioactivity data into the MN. Each .mzXML file was given the corresponding fraction name (F1—F11). The bioactivity was classified into three groups: IC_50_ < 10 μg/mL in spike assay = active, IC_50_ from 10 to 50 μg/mL in spike assay = moderately active, IC_50_ > 50 μg/mL in spike assay or % of caspase inhibition <50% at 50 μg/mL = inactive. Fractions F2 and F3 were attributed as active, F4—F9 were determined as moderately active and F1, F10 and F11 were classified as inactive. The metadata table was saved as .txt file.

### 3.9 Molecular network generation parameters

A feature-based MN was generated on the GNPS website with the following settings: parent and fragment ion mass tolerance = 0.02 Da, cosine score = 0.6, network topK = 10, minimum matched fragment ions = 4, maximum connected component size = 100, maximum shift between precursors = 500 Da (Wang et al., 2016). The network was searched against the GNPS spectra library. For a spectra library annotation, six peaks must match between a network and a library spectrum with a cosine score above 0.7. For quantification, row sum normalization and the summarization of all files within a group were selected. The obtained MN can be accessed by the following address: https://gnps.ucsd.edu/ProteoSAFe/status.jsp?task=104b80cc65e44c8c86e6a86b255b2045. The MN was visualized with Cytoscape 3.8.2 (Shannon et al., 2003).

### 3.10 Cells and virus

Caco-2-F03 cells from the Resistant Cancer Cell Line Colletion were grown at 37°C in minimal essential medium (MEM) supplemented with 10% fetal bovine serum (FBS), 100 IU/mL penicillin and 100 μg/mL streptomycin. All culture reagents were purchased from Sigma-Aldrich. The SARS-CoV-2 variant (SARS-CoV-2/FFM7, MT358643) used in the experiment was isolated from Caco-F03 cells. SARS-CoV-2 stocks were cultivated for a maximum of three passages and stored at −80°C.

### 3.11 Pre-screening assay

A phenotypic screening platform using the measurement of caspase 3/7 activation to monitor virus infection was utilized as previously described (Bojkova et al., 2023). Briefly, Caco-2-F03 cells were seeded into 96-well plates (50,000 cells/well) and incubated at 37°C for 4 days. After the cells reached confluence, the supernatant was replaced by 25 μL/well of fresh medium, 25 μL/well of medium containing tested samples (50 μg/mL) in singlets, and 50 μL/well SARS-CoV-2 suspension (MOI 0.01). Remdesivir (10 μM) was used as positive control. After infection of Caco-2-F03 cells and incubation for 48 h, the caspase 3/7 inhibition was measured using the Caspase-Glo assay kit (Promega), according to the manufacturer’s instructions. Briefly, 100 µL of Caspase-Glo reagent were added to each well, mixed, and incubated at room temperature for 30 min. Luminescence intensity was measured using an Infinite M200 microplate reader (Tecan). The raw values were subtracted by the cell control (no drug, no virus). The final results are expressed as percentage of inhibition relative to the virus control which received no treatment.

### 3.12 Spike immunostaining assay

Caco-2-F03 cells were seeded into 96-well plates (50,000 cells/well) and incubated at 37°C for 4 days. After the cells reached confluence, the supernatant was replaced by 25 μL/well of fresh medium, 25 μL/well of medium containing serially diluted fractions or pure compounds in triplicates, and 50 μL/well SARS-CoV-2 suspension (MOI 0.01). Remdesivir was used as positive control. 24 h post infection, cells were fixed with acetone:methanol (40:60) solution and immunostaining was performed using a monoclonal antibody directed against the spike protein of SARS-CoV-2 (1:1,500, Sinobiological), which was detected with a peroxidase-conjugated anti-rabbit secondary antibody (1:1,000, Dianova), followed by addition of AEC substrate. The S positive area was scanned and quantified by the Bioreader 7000-F-Z-I microplate reader (Biosys). The results are expressed as percentage of inhibition relative to virus control which received no drug.

### 3.13 Cytotoxicity

The cytotoxicity of tested fractions and pure compounds was determined in parallel to the spike immunostaining assay. The supernatant of confluent Caco-2-F03 was replaced by 75 μL/well of fresh medium and 25 μL/well of medium containing serially diluted fractions and pure compounds in triplicates and incubated for 24 h. Cell viability was measured by 3-(4,5-dimethylthiazol-2-yl)-2,5-diphenyltetrazolium bromide (MTT) dye reduction assay. 25 μL of MTT solution (2 mg/mL in PBS) were added per well, and the plates were incubated at 37°C for 4 h. Afterwards, the cells were lysed using 100 μL of a buffer containing 20% SDS and 50% N,N-dimethylformamide with the pH adjusted to 4.7 at 37°C for 4 h. Absorbance was determined at 560 nm (reference wavelength 620 nm) using a Tecan infinite M200 microplate reader (TECAN).

## Data Availability

The original contributions presented in the study are included in the article/Supplementary Material, further inquiries can be directed to the corresponding author.
