# Supplementary material for: Discovery of anti-SARS-CoV-2 secondary metabolites from the heartwood of Pterocarpus santalinus using multi-informative molecular networking
Source: Front Mol Biosci. 2023 Jun 6;10:1202394. doi: 10.3389/fmolb.2023.1202394 (PMC10280016; doi:10.3389/fmolb.2023.1202394)
Supplement: Supplementary file 1 [file DataSheet1.PDF]

SUPPORTING INFORMATION

Discovery of anti-SARS-CoV-2 secondary metabolites from the heartwood of *Pterocarpus santalinus* using multi-informative molecular networking

Andreas Wasilewicz,[1,2] Julia Zwirchmayr,[1] Benjamin Kirchweger,[1] Denisa Bojkova,[3] Jindrich Cinatl Jr.,[3] Holger F. Rabenau,[3] Judith M. Rollinger,[1] Mehdi A. Beniddir,[4] Ulrike Grienke[1]*

* Correspondence

Ulrike Grienke
ulrike.grienke@univie.ac.at

1 Department of Pharmaceutical Sciences, Division of Pharmacognosy, Faculty of Life Sciences, University of Vienna, Josef-Holaubek-Platz 2, 1090 Vienna, Austria

2 Vienna Doctoral School of Pharmaceutical, Nutritional, Sport Sciences, University of Vienna, Josef-Holaubek-Platz-2, 1090 Vienna, Austria

3 Institute of Medical Virology, University Hospital Frankfurt, Paul-Ehrlich-Straße 40, 60596 Frankfurt am Main, Germany

4 Équipe Chimie des substances naturelles, BioCIS, CNRS, Université Paris-Saclay, 17, avenue des Sciences, 91400, Orsay, France

Table of content

Figure S1. Collective TLC analysis of PS-DE and F1 – F11. 3

Figure S2. UHPLC-MS TIC of F1 – F6. 4

Figure S3. UHPLC-MS TIC of F7 – F11. 5

Figure S4. UPLC-ELSD chromatograms of PS-DE and fractions F1 – F5. 6

Figure S5. UPLC-MS chromatograms of PS-DE and fractions F1 – F5. 7

Figure S6. UPLC-ELSD chromatograms of fractions F6 – F11. 8

Figure S7. UPLC-MS chromatograms of fractions F6 – F11..9

Table S1. Analyzed Nodes from the Molecular Network. 10

Table S2. Yields of Fractions F1 – F11. 11

SARS-CoV-2 M^{pro} inhibitory activity of compound **5**. 11

Figure S8. 3D structural alignment of compounds **5** (cyan) and **22** (orange). 12

Table S3. Solvent Ratio (v/v/v/v) of HEMWat Systems used in this Study...12

Figure S9. Fractionation tree of PS-DE. 13

Figure S10. ^{1}H NMR (500 MHz, $CDCl_3$) spectrum of compound **9**. 14

Figure S11. ^{13}C APT NMR (125 MHz, $CDCl_3$) spectrum of compound **9**. 15

Figure S12. HSQC NMR (500 MHz, $CDCl_3$) spectrum of compound **9**. 16

Figure S13. HMBC NMR (500 MHz, $CDCl_3$) spectrum of compound **9**. 17

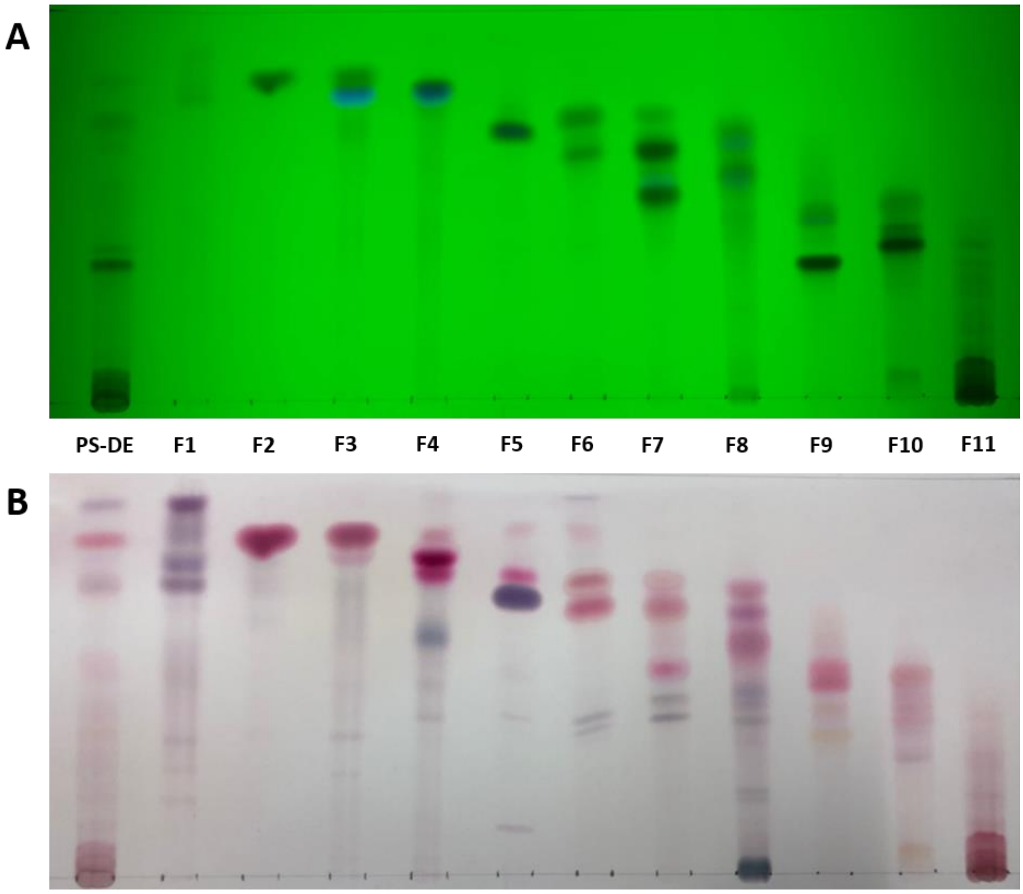

Figure S2. Collective TLC analysis of PS-DE and F1 – F11. Detection at (A) UV 254 nm and (B) visible light after derivatization with vanillin (1%)/sulfuric acid (5%).

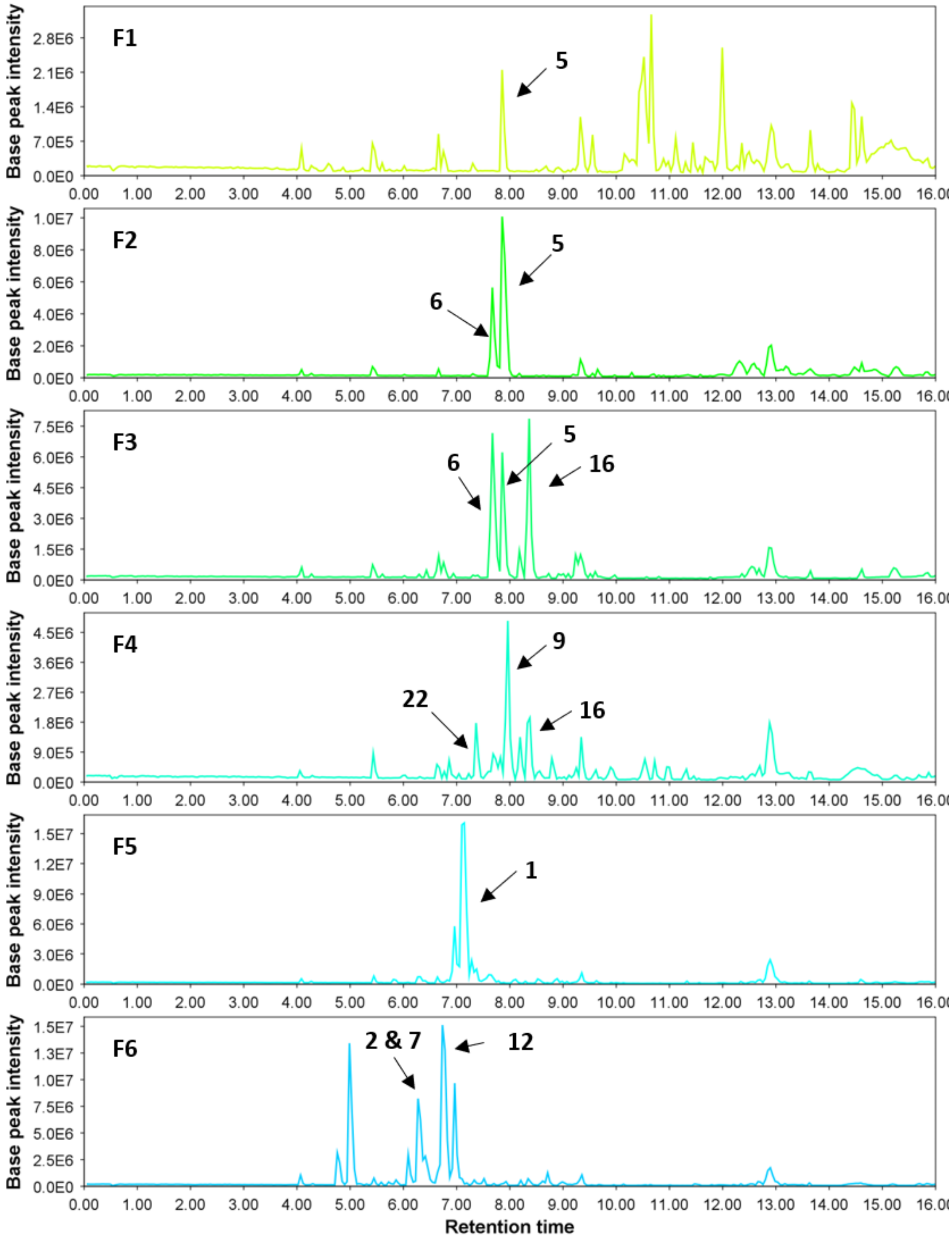

Figure S2. UHPLC-MS TIC of F1 – F6.

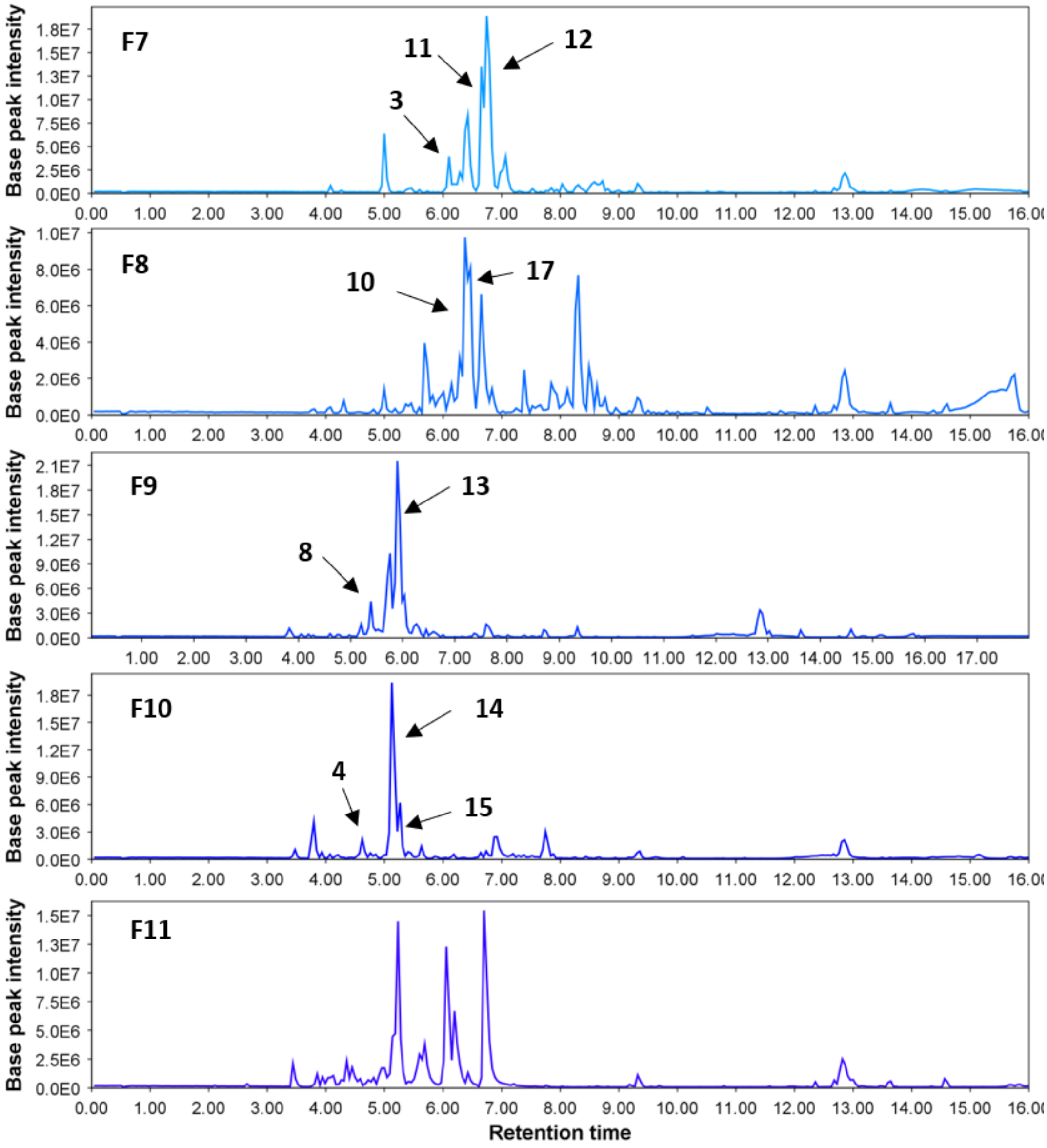

Figure S3. UHPLC-MS TIC of F7 – F11.

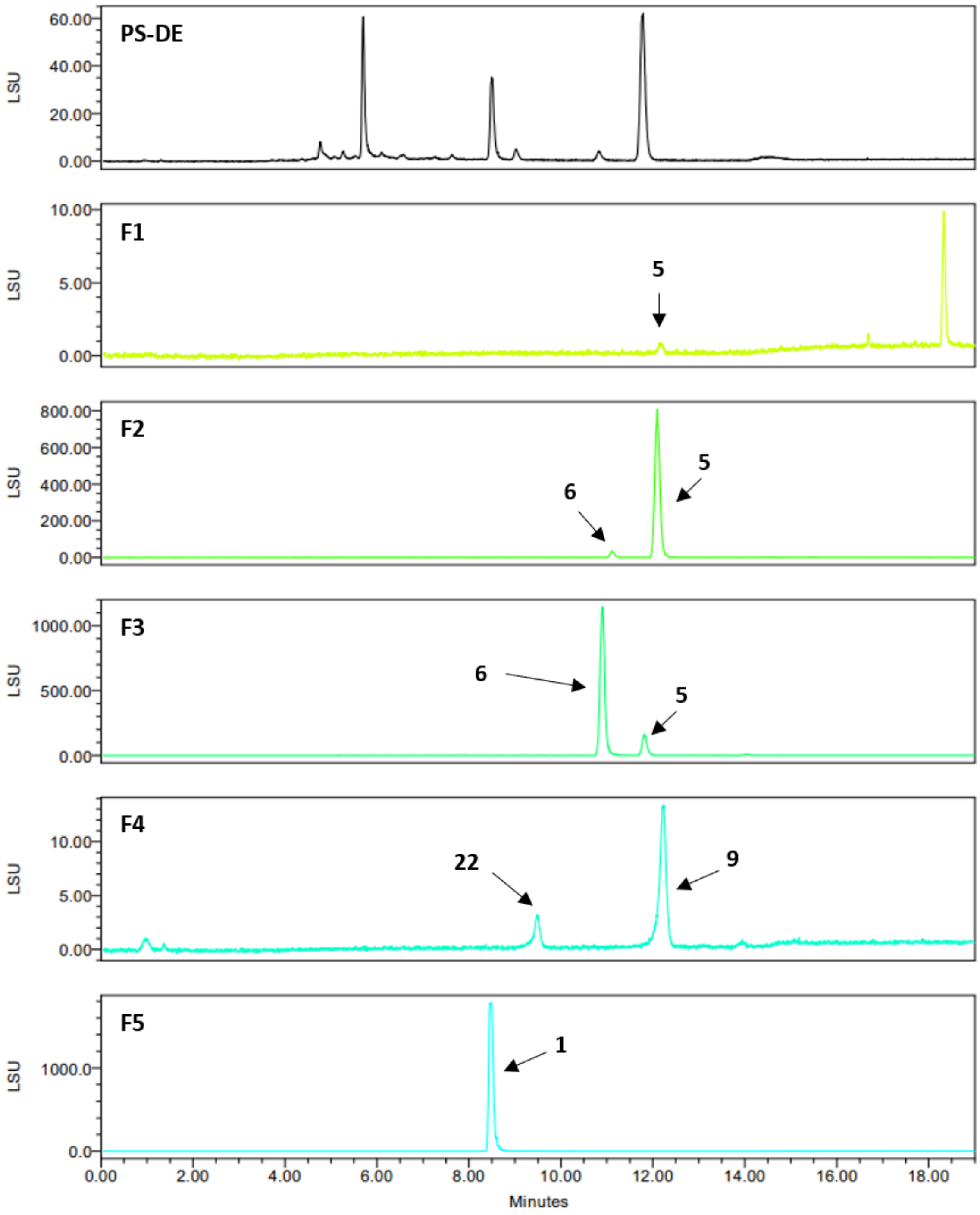

Figure S4. UPLC-ELSD chromatograms of PS-DE and fractions F1 – F5.

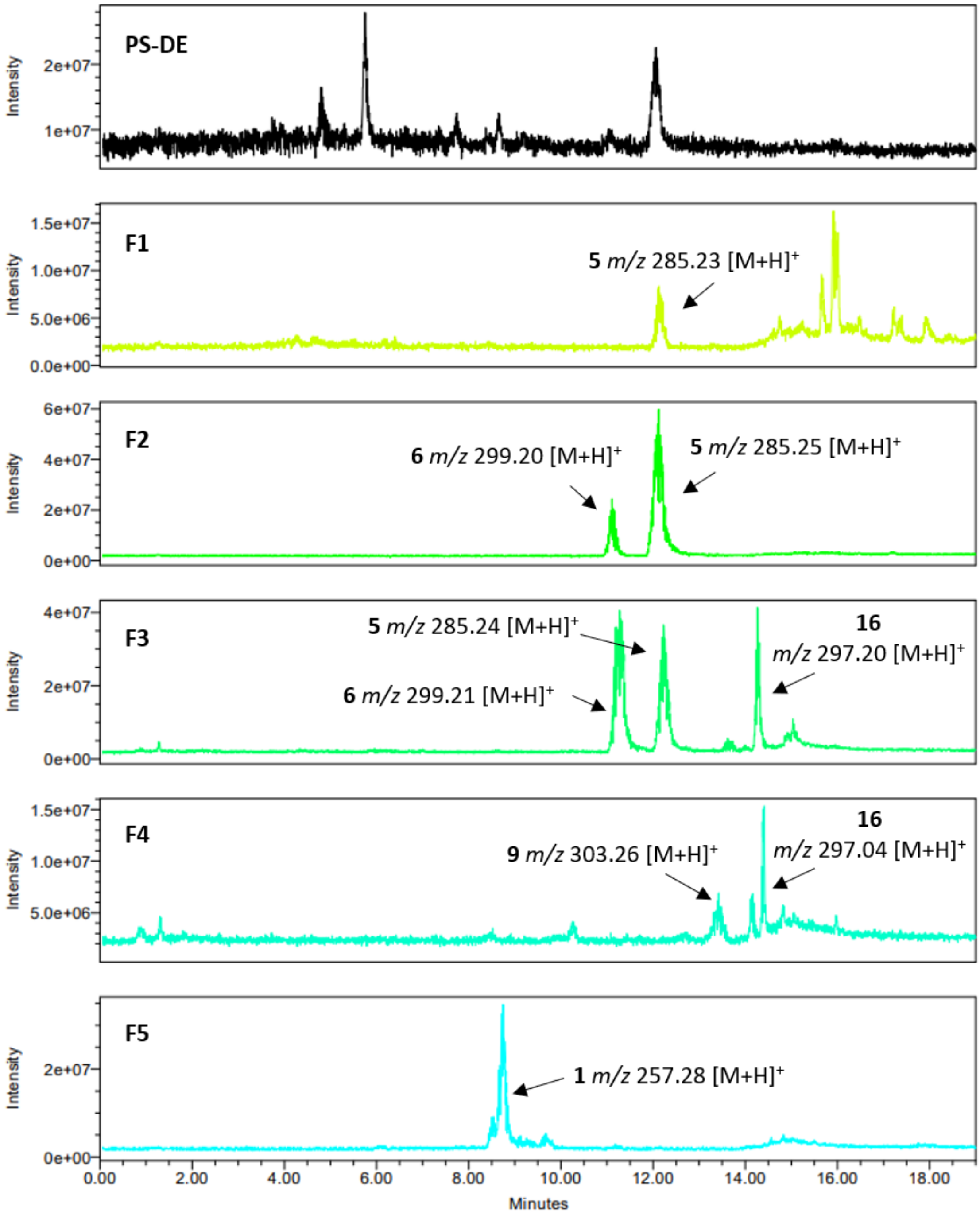

Figure S5. UPLC-MS chromatograms of PS-DE and fractions F1 – F5.

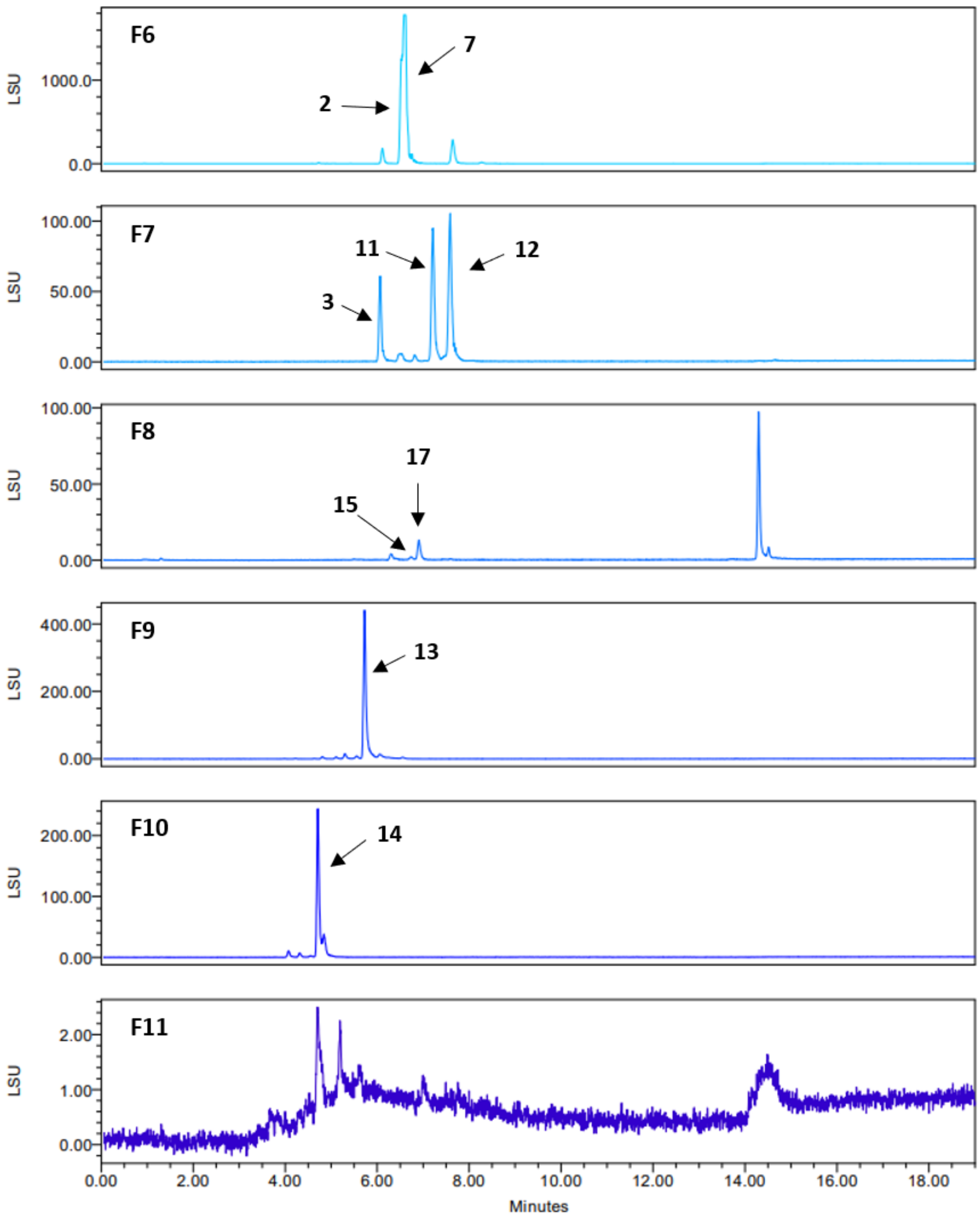

Figure S6. UPLC-ELSD chromatograms of the fractions F6 – F11.

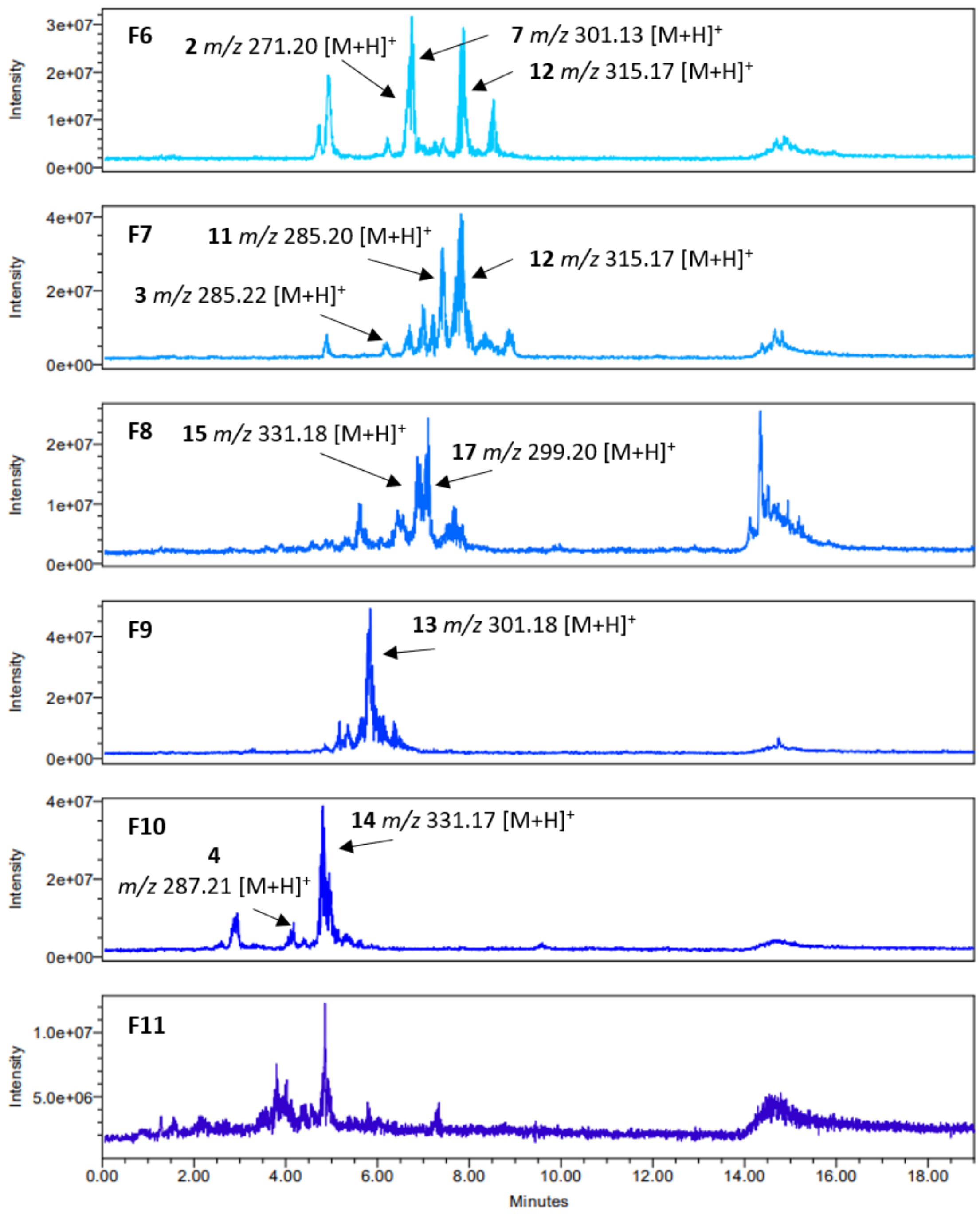

Figure S7. UPLC-MS chromatograms of fractions F6 – F11.

Table S1. Analyzed nodes of the Molecular Network.

molecular family	*m/z*[a,b]	retention time (RT)[b] [min]	molecular formula	GNPS annotation	confirmed structure (**compound number**)
MF-A					
	257.1172	7.15	$C_{16}H_{16}O_3$	pterostilbene	(*E*)-pterostilbene (**1**)
MF-B					
	271.0966	6.32	$C_{16}H_{14}O_4$	-	(-)-medicarpine (**2**)
	285.0753	6.10	$C_{16}H_{12}O_5$	-	(-)-maackiain (**3**)
	285.1122	7.85	$C_{17}H_{16}O_4$	homopterocarpin	(-)-homopterocarpin (**5**)
	287.0912	4.62	$C_{16}H_{14}O_5$	-	(-)-3,8-dihydroxy-9-methoxypterocarpan (**4**)
	299.0912	7.67	$C_{17}H_{14}O_5$	-	(-)-pterocarpin (**6**)
	301.1074	6.27	$C_{17}H_{16}O_5$	-	(-)-8-hydroxyhomo-pterocarpan (**7**)
	301.1592	7.96	$C_{18}H_{22}O_4$	-	pterosantalin D (**9**)
	331.1177	6.34	$C_{18}H_{18}O_6$	-	(-)-pterosantalin E (**10**)
	333.0970	5.40	$C_{17}H_{16}O_7$	-	(-)-pterosantalin C (**8**)
	347.1130	6.96	$C_{18}H_{18}O_7$[c]	-	-
MF-C					
	269.0811	5.70	$C_{16}H_{12}O_4$	-	-
	285.0761	6.66	$C_{16}H_{12}O_5$	7-*O*-methylgenistein	7-*O*-methylgenistein (**11**)
	297.0758	8.36	$C_{17}H_{12}O_5$	-	3,9-*O*-dimethylcoumestrol (**16**)
	299.0915	6.30	$C_{17}H_{14}O_5$[c]	-	-
	299.0916	5.82	$C_{17}H_{14}O_5$[c]	-	-
	299.0916	6.44	$C_{17}H_{14}O_5$	-	pterosantalin F (**17**)
	301.0706	5.91	$C_{16}H_{12}O_6$	-	santal (**13**)
	315.0866	6.77	$C_{17}H_{14}O_6$	-	7,3′-di-*O*-methylorobol (**12**)
	331.0813	5.16	$C_{17}H_{14}O_7$	khrinone C	khrinone C (**14**)
	345.0975	6.65	$C_{18}H_{16}O_7$[c]	-	-
	361.0918	5.24	$C_{18}H_{16}O_8$	-	pterosantalin B (**15**)
MF-D					

	583.1602	5.23	$C_{33}H_{26}O_{10}$[c]	-	-
	597.1755	4.95	$C_{34}H_{28}O_{10}$[c]	-	-
	597.1761	6.06	$C_{34}H_{28}O_{10}$[c]	-	-
	611.1191	5.60	$C_{35}H_{30}O_{10}$[c]	-	-
MF-E					
	541.2222	8.31	$C_{30}H_{20}O_{10}$[c]	-	-

[a] all *m/z* ratios present $[M+H]^+$ adducts; [b] data were obtained from UHPLC-MS analysis; [c] putatively determined.

Table S2. Yields of Fractions F1 – F11.

Fraction	F1	F2	F3	F4	F5	F6	F7	F8	F9	F10	F11
Yield [mg]	24.9	83.3	5.5	4.9	41.8	13.8	12.5	9.3	54.4	14.8	271.6

SARS-CoV-2 M^{pro} inhibitory activity of compound **5**.

Method

SARS-CoV-2 M^{pro} was produced as described in Wasilewicz et al., 2023 (J. Nat. Prod. 2023, 86, 2, 264–275). M^{pro} inhibition was tested using a FRET assay based on cleavage of a fluorescent substrate (crb1101508j, Discovery Peptides) containing an N-terminal 5-carboxyfluorescein (5-FAM) and a 4-((4-(Dimethylamino)phenyl)azo)benzoyl (DABCYL) e quencher. The assay buffer consisted of 50 mM TRIS-HCl (pH = 7,55), 1 mM EDTA and 5 mM dithiothreitol (DTT). Compounds were preincubated with M^{pro} in black flat-bottomed 96-well plates for 30 minutes at 30°C. After the addition of substrate, fluorescence was measured at 30°C with Exc/Emi 483/530 nm. 60 cycles of 30 sec were recorded using a Tecan Sparks plate reader and the initial velocities were calculated from the linear section of the reaction curves. The total assay volume per well was 100 µL with final concentrations of 50 nM M^{pro} enzyme, 10 µM substrate and 0.2% DMSO. Final test concentrations of boceprevir (positive control) and compound **5** were 20 µM and 50 µM, respectively. Each experiment was performed with three well replicates and two background ontrol wells without M^{pro} to correct for background fluorescence. The % of M^{pro} inhibition was determined by the means of three independent experiments compared to DMSO control (negative control).

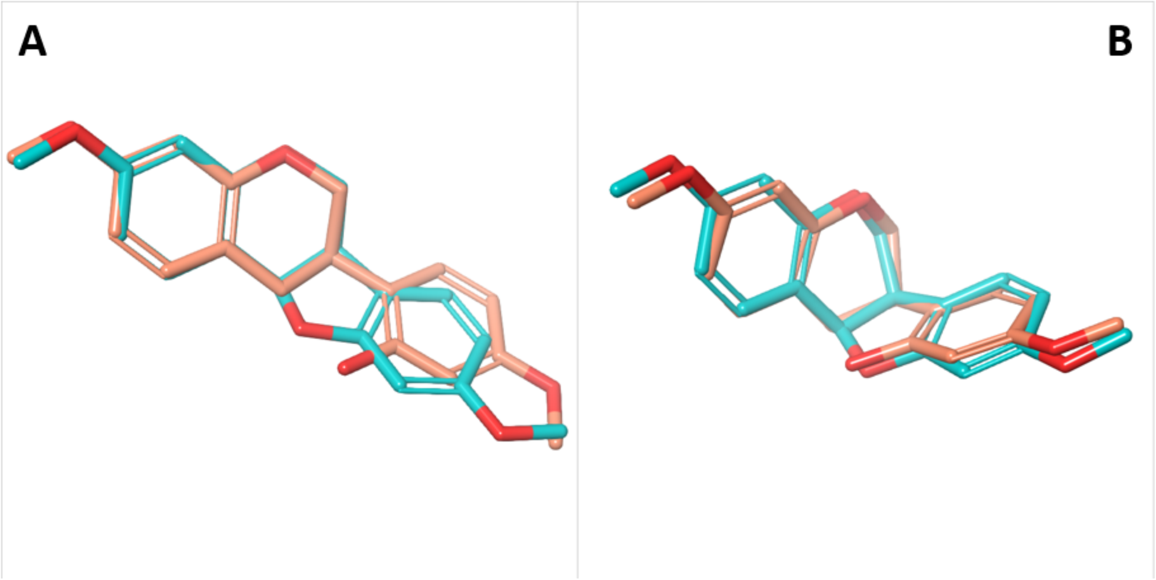

Figure S8. 3D structural alignment of compounds **5** (cyan) and **22** (orange). Due to cis-conformation, compound **5** possesses two chair conformers which can interconvert via ring-flip. Compound **22** aligns well to both conformers but shows better alignment with conformation (B). Alignment was performed using the Ligand Alignment tool from Maestro (Schödinger release 2020-3: *Maestro 12.5*; Schrödinger LLC: New York, 2020).

Table S3. Solvent Ratio (v/v/v/v) of HEMWat Systems used in this Study.

HEMWat system no.	hexane	ethyl acetate	methanol	water
23	4	1	4	1
19	3	2	3	2
18	6	5	6	5
17	1	1	1	1
16	5	6	5	6
14	1	2	1	2
13	2	5	2	5

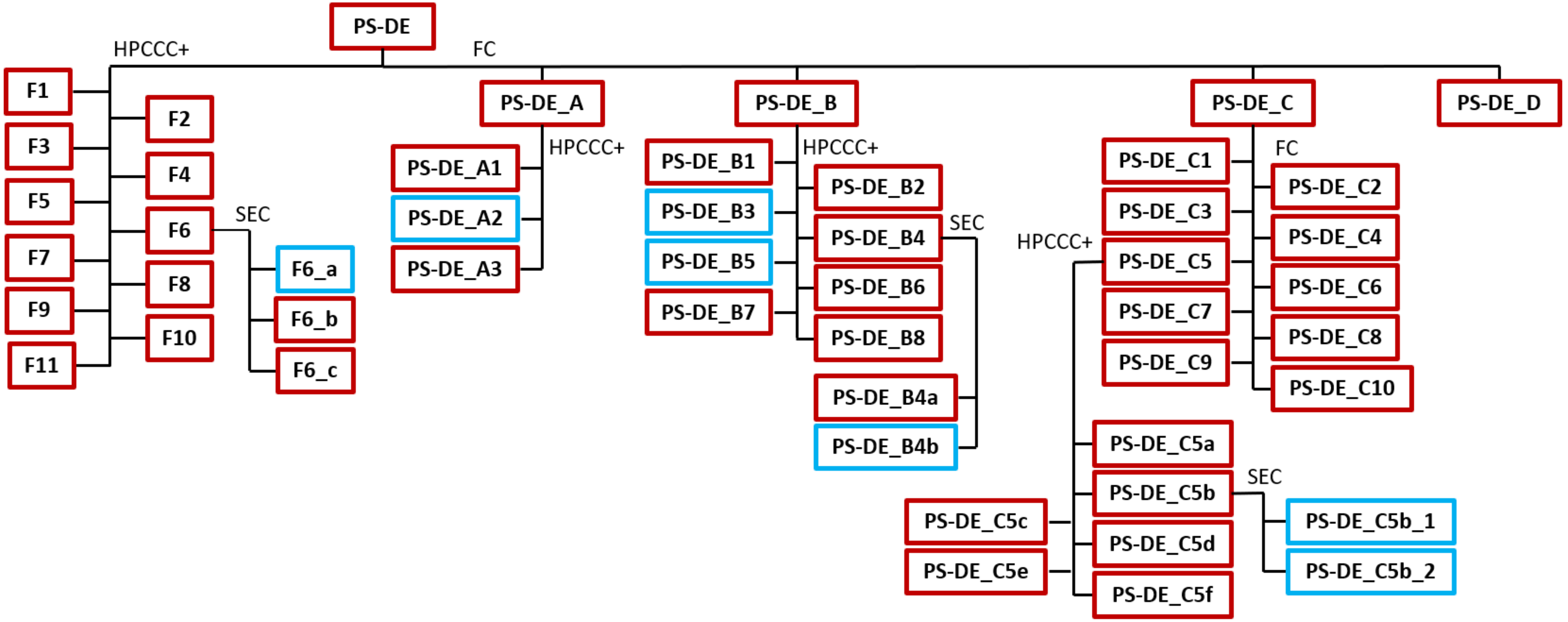

Figure S9. Fractionation tree of PS-DE. Red squares represent fractions, blue squares represent purified isolates; HPCCC+, high performance counter-current chromatography, FC, flash chromatography; SEC, Sephadex LH-20 gel filtration chromatography

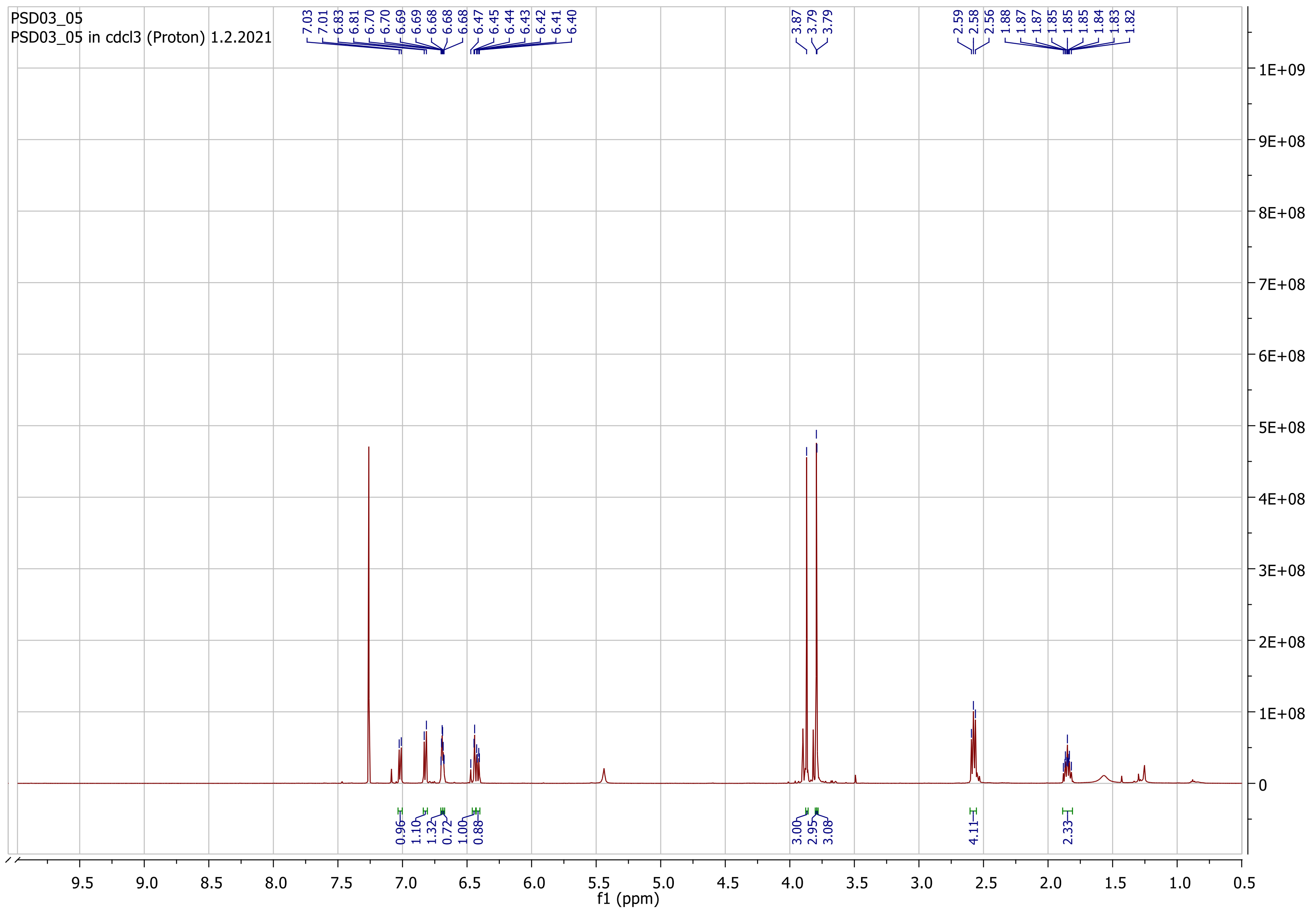

Figure S10. ^{1}H NMR (500 MHz, $CDCl_3$) spectrum of compound **9**.

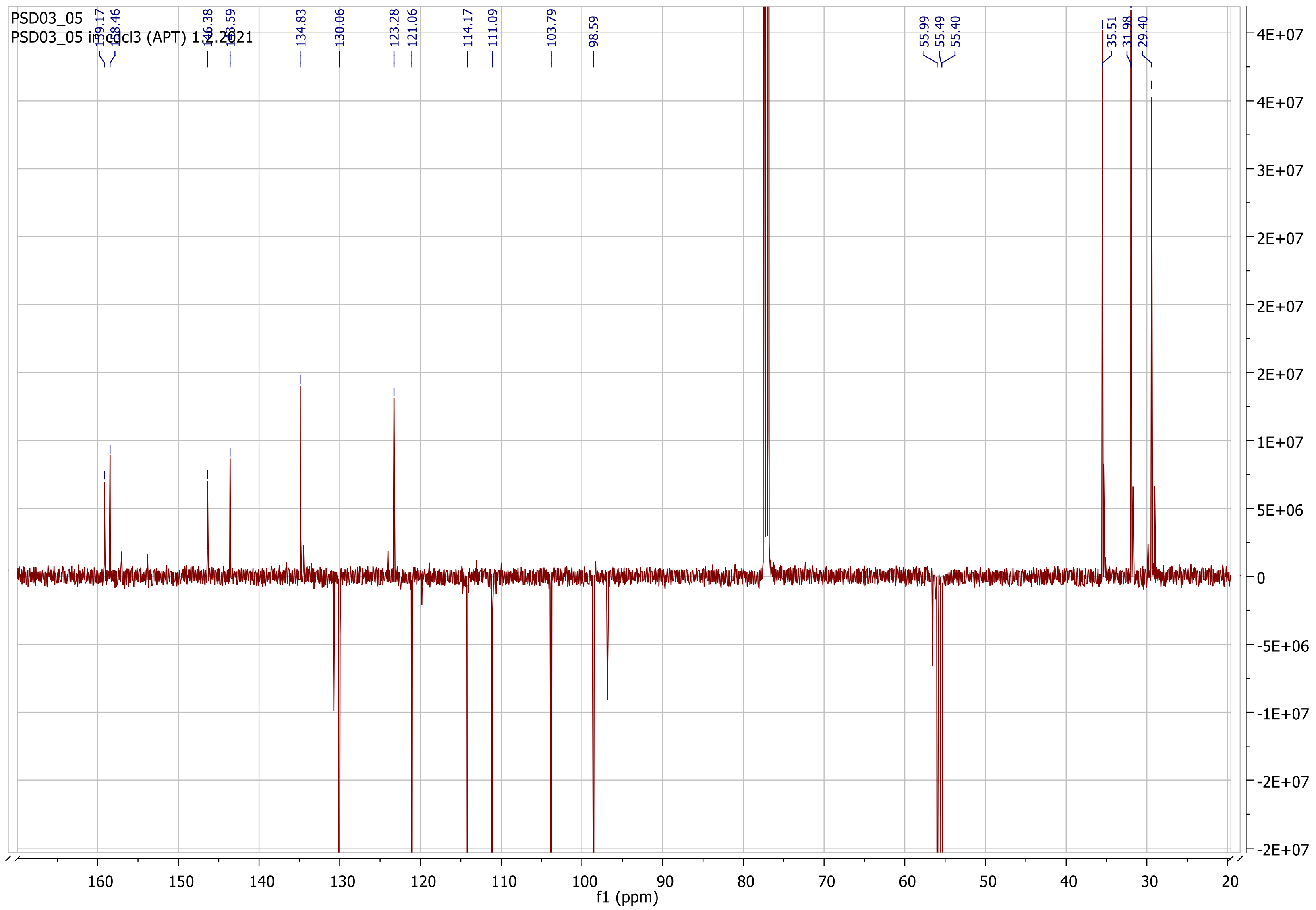

Figure S11. ^{13}C APT NMR (125 MHz, $CDCl_3$) spectrum of compound **9**.

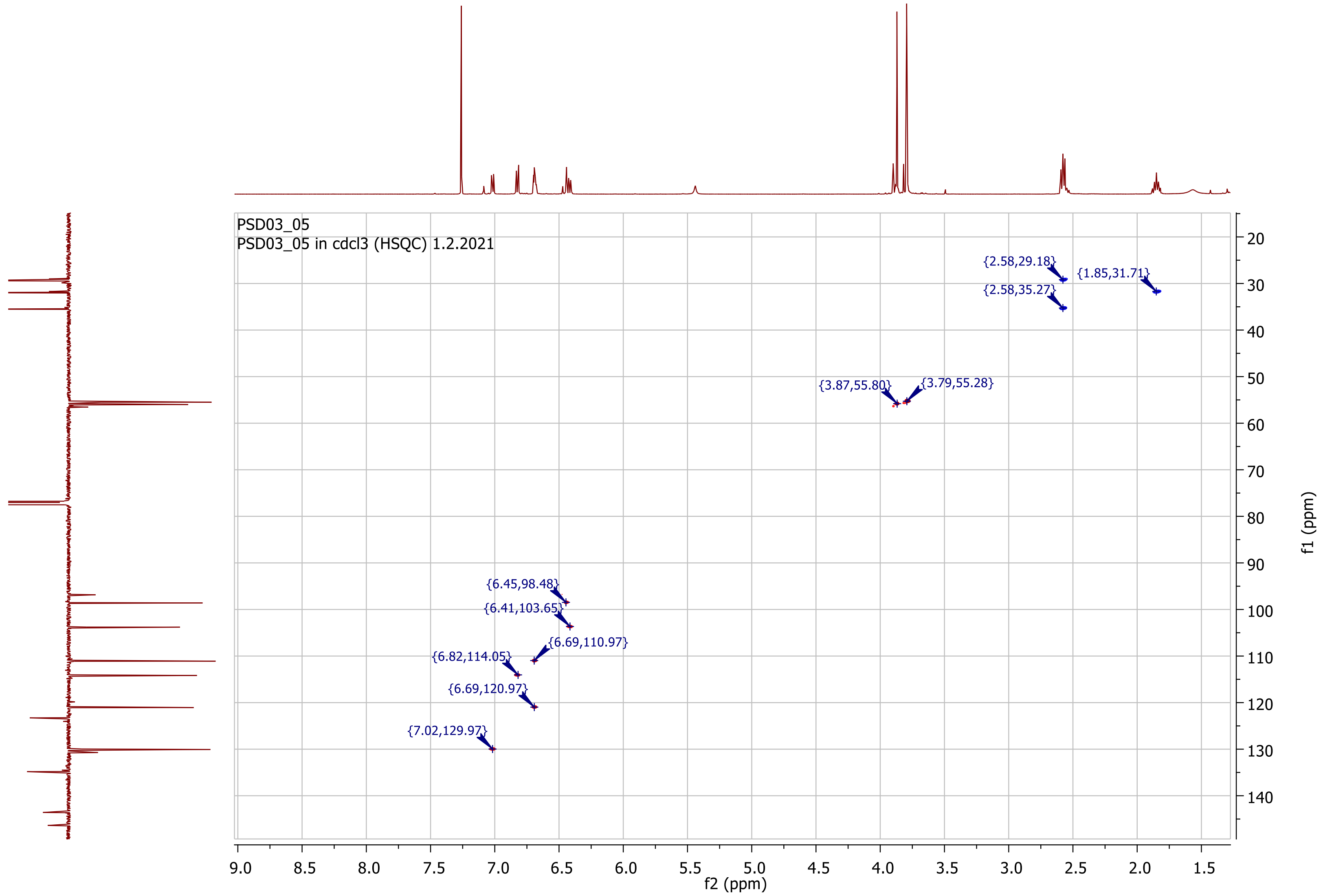

Figure S12. HSQC NMR (500 MHz, $CDCl_3$) spectrum of compound **9**.

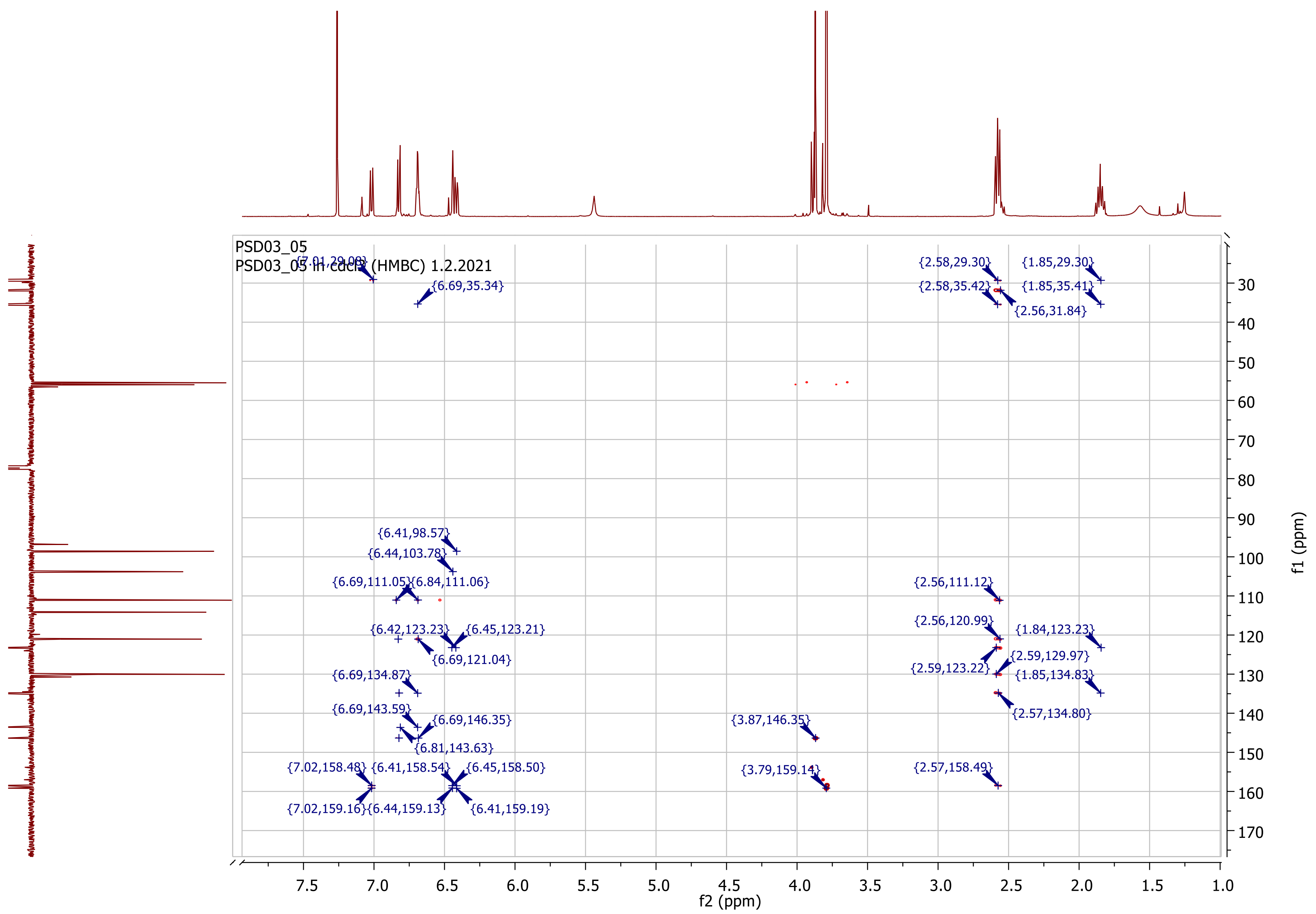

Figure S13. HMBC NMR (500 MHz, $CDCl_3$) spectrum of compound **9**.
